# Mitochondrial–Stem Cell Connection: Providing Additional Explanations for Understanding Cancer

**DOI:** 10.3390/metabo14040229

**Published:** 2024-04-17

**Authors:** Pierrick Martinez, Ilyes Baghli, Géraud Gourjon, Thomas N. Seyfried

**Affiliations:** 1 Scientific and Osteopathic Research Department, Institut de Formation en Ostéopathie du Grand Avignon, 84140 Montfavet, France; g.gourjon@ifoga.fr; 2 International Society for Orthomolecular Medicine, Toronto, ON M4B 3M9, Canada; ilyes.baghli@isom.ca; 3 Biology Department, Boston College, Chestnut Hill, MA 02467, USA; thomas.seyfried@bc.edu

**Keywords:** tumorigenesis, cancer stem cells, oxidative phosphorylation, glycolysis, glutaminolysis, tumor microenvironment, metastases

## Abstract

The cancer paradigm is generally based on the somatic mutation model, asserting that cancer is a disease of genetic origin. The mitochondrial–stem cell connection (MSCC) proposes that tumorigenesis may result from an alteration of the mitochondria, specifically a chronic oxidative phosphorylation (OxPhos) insufficiency in stem cells, which forms cancer stem cells (CSCs) and leads to malignancy. Reviewed evidence suggests that the MSCC could provide a comprehensive understanding of all the different stages of cancer. The metabolism of cancer cells is altered (OxPhos insufficiency) and must be compensated by using the glycolysis and the glutaminolysis pathways, which are essential to their growth. The altered mitochondria regulate the tumor microenvironment, which is also necessary for cancer evolution. Therefore, the MSCC could help improve our understanding of tumorigenesis, metastases, the efficiency of standard treatments, and relapses.

## 1. Introduction

Despite the therapeutic advances of the last century, inter alia, chemotherapy, radiotherapy, and immunology, cancer’s incidence and mortality continue to rise. Many theories have been proposed to explain the origin of cancer [1]. In the 1920s, the mitochondrial metabolic theory suggested that cancer is caused by a disorder in cellular respiration [2,3]. A few decades later, the discovery of deoxyribonucleic acid (DNA) led to the somatic mutation theory (SMT), hypothesizing that cancer results from an abnormal proliferation of cells generated by the accumulation of mutations (DNA) within a single cell [4]. Another more recent model is the cancer stem cells (CSCs) theory. In the latter model, the CSCs are a small subpopulation of cells within tumors that possess the ability to self-renew, differentiate, and form tumors [5]. Finally, the tissue organization field theory of cancer (TOFT) suggests that cancer is a tissue-based disease and arises in disorganized tissue and not in individual cells [6]. Nowadays, the SMT is the most widespread theory.

It is generally accepted that the environmental and biopsychosocial factors (ecosystem, lifestyle, work environment, home environment, drugs, etc.) play an important role in tumorigenesis [7]. For instance, many traditional communities that follow a hunter–gatherer diet and do not live in industrialized regions have a lower incidence of cancer [8]. Tumorigenesis observed in animal models provides a basis for understanding the mechanisms behind it. Generally, five methods are used to generate tumors: spontaneous mutations, chemical agents/radiation, retroviruses, DNA microinjections, and local or systemic injection of malignant cells [9]. Cancer induction by chemical agents or ionizing radiation is far more effective than by other methods [10]. Yet, the somatic mutation theory may not provide a complete explanation of cancer. For example, mutations in primordial genes have been observed in healthy patients, and a few substances can be carcinogenic without being mutagenic. In the 1920s, Warburg developed an understanding of cancer based on mitochondrial alterations. A few decades later, the introduction of the concept of CSCs furthered the comprehension of cancer development. Our approach aims to combine the two in order to provide supplementary insights into the different stages of cancer development, specifically mitochondrial alterations in CSCs being able to reduce OxPhos efficiency.

## 2. The Somatic Mutation Model Alone May Not Explain Tumorigenesis

The Somatic Mutation Model asserts that a mutated gene is responsible for tumorigenesis. However, there is no evidence whatsoever that identified DNA mutations cause a cell to go from normal to malignant [11,12]. All the cellular events and rearrangements that lead to malignancy remain elusive [13] particularly in the early stages of tumorigenesis [14]. Because of this, in most cases, identifying a specific mutation that initiates tumorigenesis is impossible [15]. Morales et al. [16] have shown that modification of HRAS gene increases fibroblast telomerase activity, making cells immortal but not causing tumorigenesis. Loss of oncogene expression may not be correlated with suppression of tumorigenesis, suggesting that oncogenes may be an effect rather than a cause of cancer [17]. A recent analysis of the data shows that almost all genes could be associated with cancer [18]. Tumor DNA sequencing data have shown that cancer cells have multiple heterogeneous DNA mutations [11]. Mutations may even be heterogeneous between tumors within the same cancer category [19]. A tumor is genomically heterogeneous, with each cell having a different mutational signature [20]. The SMT theory suggests that tumorigenesis arises from a series of somatic mutations, perhaps at key points in the genome. This would result in a pattern of mutations shared by the genomes of all cancer cells. However, the observed heterogeneity invalidates the existence of a mutational pattern. Considering that somatic mutations may be a consequence rather than a cause, genome heterogeneity would be better explained by combining SMT with other factors described in the MSCC theory. It becomes difficult to determine a causal mechanism with SMT concept, especially with tumors where we can find more than 10,000 mutations in tumor cell gene exons [21]. On the other hand, many genes defined as cancer driver genes have been found in normal, healthy cells [22,23,24,25,26,27,28]. These mutations even appear in important genes like KRAS and even TP53, known as the “guardian of the genome” [29,30]. The TP53 gene also appears mutated in diseases other than cancer, such as rheumatoid arthritis [31,32,33]. The most frequently mutated gene, TP53, appears mutated in only 35% of all cancers [34]. Moreover, some carcinogens do not always cause mutations [35,36,37] and this seems to contradict the theory. Non-genotoxic carcinogens could cause cancer by modifying the endocrine system, inducing cytotoxicity, increasing inflammation, suppressing the immune system, and causing oxidative stress [36]. Several inert substances, such as plastics, when implanted in the tissues of sensitive animal hosts, induced tumors locally [38]. For example, substances such as methapyrilene, hexachlorobenzene, chloroform, p-dichlorobenzene, and d-limonene could result in a tumor even if they do not release genotoxic compounds that induce mutations in candidate genes (oncogenes, tumor suppressor genes) [39]. Overall, the criteria for establishing a causal link between the somatic mutation theory and cancer are rarely met [40]. Therefore, in the light of the evidence provided, somatic mutation could be a consequential and subsequent phenomenon occurring after the onset of tumorigenesis [41].

## 3. Role of Mitochondria in Tumorigenesis

Mitochondria are complex organelles that influence cancer initiation, growth, survival, and metastasis [42,43]. Known carcinogens can cause mitochondrial dysfunction, especially chemical agents/drugs [44,45,46], radiation [47], and viruses [48]. Thus, mitochondrial damage may precede tumorigenesis [49]. Mitochondrial dysfunction is associated with altered metabolisms and can promote tumorigenesis as well as metastases [50]. Mitochondrial DNA (mtDNA) mutations have been reported in all cancer types [51,52] and are a major source of driver mutation in cancer [53,54]. However, like somatic mutations, it is not sufficient enough to explain all cancers. Indeed, no pathogenic mtDNA mutations were found in five mouse brain tumors [55]. So, it seems that genetic mutations are not the cause of cancer, even in mitochondria, unless they can cause a chronic reduction in the efficiency of oxidative phosphorylation (OxPhos) [56]. Cancer may result from progressive disruption of adenosine triphosphate (ATP) synthesis by OxPhos, leading to compensatory ATP synthesis [3]. Pedersen synthesized data from over twenty studies showing that no highly malignant tumor had normal mitochondria in number and morphology. The total number of mitochondria in tumor cells was significantly lower than in healthy cells, and the total respiratory capacity of tumor mitochondria was lower than in healthy cells [57]. The degree of malignancy could be directly correlated with the degree of mitochondrial structural abnormalities [58]. It has been shown that human cancers exhibiting structural alterations in mitochondria cristae can suffer from swelling and partial or total cristolysis [59,60]. Evidence suggests that various cancers exhibit abnormalities in mitochondrial number, structure, or function [61]. The quantity of altered mitochondria changes depending on the type of cancer and their need to grow [62]. Disruption of OxPhos leads to the accumulation of mutagenic and carcinogenic reactive oxygen species (ROS). Cancer cells have higher levels of ROS than healthy cells due to mitochondrial dysfunction [63]. Exogenous ROS sources produce oxidative stress, which leads to mitochondrial dysfunction, resulting in increased endogenous ROS production and potentially tumorigenesis [64]. In the 1920s, Warburg was the first to argue that cancer was initiated by damaged cellular respiration [2,65]. As nuclear mutations occur, mutations in mtDNA are considered secondary risk factors and can only be linked to the origin of cancer if they also disrupt OxPhos function [56]. However, OxPhos is rarely normal in cells containing many mtDNA mutations, which is the case for many cancers [52]. Somatic mutations can only be due to genotoxic carcinogens, limiting the possibility of non-genotoxic agents causing tumors, as explained by SMT. Conversely, OxPhos inhibition or suppression can result from both genotoxic carcinogens [46,66] and non-genotoxic carcinogens [67,68], allowing us to better understand why these two types of compounds can cause cancer. Warburg also noted that acute damage to respiration is more likely to cause cell death [2]. Indeed, a chronic effect of radiation will lead to a decrease in OxPhos activity and ATP production compared to an acute effect [66]. Although mitochondrial mutation is not essential for tumorigenesis, chronic OxPhos insufficiency appears to be.

## 4. Nuclear Genetic Mutation Versus Mitochondrial Alteration in Tumorigenesis: Experimental Studies

**Figure 1:**
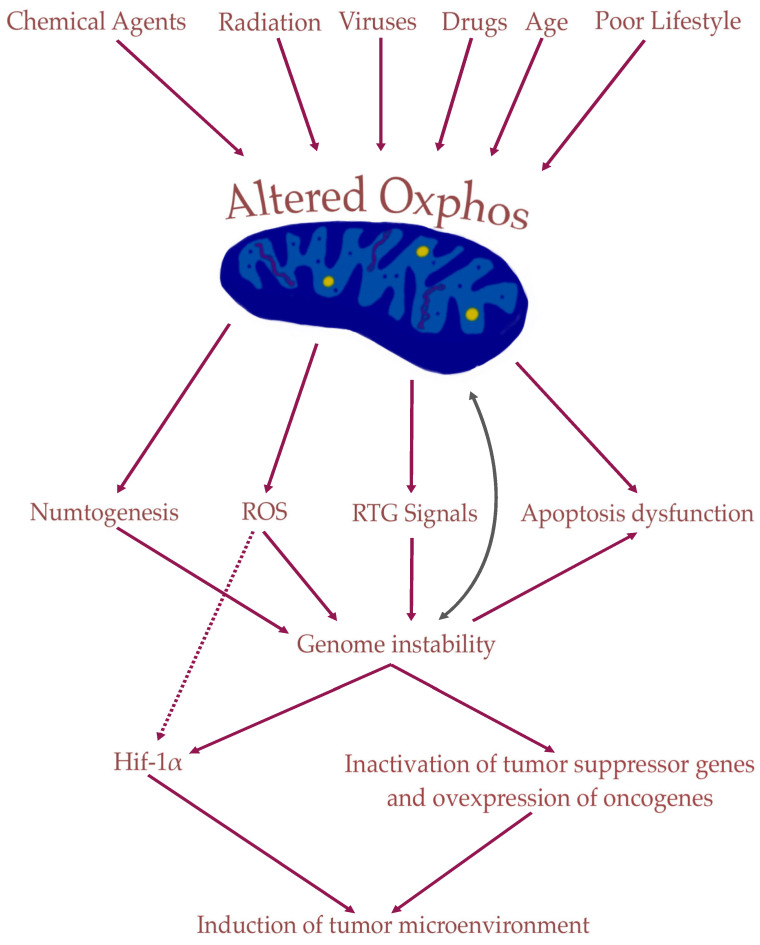
Dysfunctional mitochondria drive the alterations seen in the nuclear genome. Insufficient OxPhos drives alteration of the nuclear genome through three pathways: Numtogenesis, ROS, and RTG Signals. As mitochondria control apoptosis, apoptosis dysfunction would be an expected outcome after insufficient OxPhos. HIF-1α—Hypoxia-Inducible Factor 1α; OxPhos—Oxidative Phosphorylation; ROS—Reactive Oxygen Species; RTG—ReTroGrade.

Insufficient OxPhos would be responsible for most of the nuclear genomic changes in cancer, rather than the opposite [3]. Roskelley et al. [69] demonstrated that OxPhos was either defective or insufficient in all cancer cells and that the genome stability depended on OxPhos. Nuclear genomic modifications can occur via three pathways following mitochondrial dysfunction. The first is retrograde (RTG) signaling, which is the specific term for mitochondrial signaling to the nucleus. It involves cellular responses to changes in the state of mitochondria [70]. RTG1 and RTG3 proteins can individually enter the nucleus and modify genome expression [3,71,72]. Chronic mitochondrial stress, also known as mitostress, leads to RTG signals that create instability, overexpression of oncogenes, and inactivation of tumor suppressor genes. The second phenomenon, called numtogenesis, involves the transfer of mtDNA (or less specifically the transfer of mitochondrial components) into the nuclear genome [73]. Mitostress can lead to mitochondrial dysfunction, which in turn can lead to mitochondrial DNA transfer into the nucleus, resulting in genome instability [62,74,75]. The final pathway involves ROS. Bartesaghi et al. [76] have shown that the inhibition of mitochondrial metabolism leads to p53 mutation via a mechanism involving ROS in CSCs. Chandra et al. [77] confirmed that mitochondrial dysfunction induces genetic instability of the nuclear genome. The mechanisms that can generate cancer are summarized in Figure 1 below:

RTG signals, numtogenesis, and persistent ROS lead to the expression of numerous oncogenes and the inactivation of tumor suppressor genes, which will allow the development of the tumor environment and abnormal energy metabolism. Chronic mitostress can lead to the alteration of other cellular mitochondria, further reducing cellular respiratory capacity.

**Figure 2:**
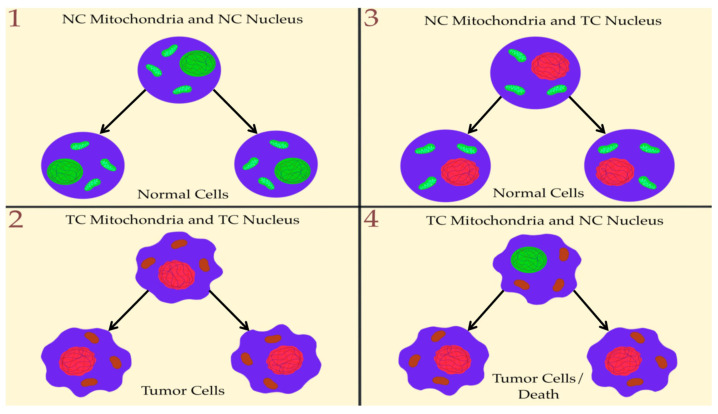
Nuclear genetic mutation versus mitochondrial dysfunction. Normal cell mitochondria and a normal cell nucleus are depicted in green, with mitochondrial and nuclear morphology indicative of normal respiration and nuclear gene expression, respectively. Tumor cell mitochondria and tumor cell nuclei are depicted in red with abnormal mitochondrial and nuclear morphology indicative of abnormal respiration and genomic instability. (**1**) Normal cell (NC) mitochondria and normal cell (NC) nucleus beget normal cells. (**2**) Tumor cell (TC) mitochondria and tumor cell (TC) nucleus beget tumor cells. (**3**) Delivery of a TC nucleus into an NC cytoplasm (with NC mitochondria) begets normal cells despite the persistence of tumor-associated genomic abnormalities. (**4**) Delivery of an NC nucleus into a TC cytoplasm (with TC mitochondria) begets tumor cells or dead cells but not normal cells. This image was extracted and reproduced with a new design with the agreement of Seyfried [3].

Studies both in vitro and in vivo have shown that tumorigenicity is suppressed when the cytoplasm of non-tumorigenic cells (containing normal mitochondria) is combined with tumor cell nuclei [78,79,80,81,82,83,84,85,86]. Nucleus genetic mutations would not be sufficient, as suggested by repeated cases where cells from various cancer types were normalized when placed in a normal environment [87,88,89]. Introducing normal mitochondria into highly malignant cells could reverse malignancy and down-regulate several oncogenic pathways. The opposite effect was produced when tumor mitochondria were transferred into the cytoplasm of normal cells [58,90]. These results further suggest that tumorigenesis depends more on mitochondrial dysfunction than on the presence of mutations in the nucleus. In contrast to normal mitochondria’s suppressive effects on tumorigenicity, tumorigenicity is enhanced when the nuclei of nontumorigenic cells are combined with tumor cells’ cytoplasm or with altered mitochondria [91,92]. All this information is summarized below in Figure 2:

These observations are consistent with Darlington’s original view that tumor cells arise from defects in the cytoplasm rather than defects in the nucleus [93]. Additionally, recent studies show that the injection of normal mitochondria in tumor cells can negatively regulate several oncogenic pathways and abnormal growth of glioma, melanoma, and metastatic breast cancer cells [90,94,95,96,97]. Cuezva and Ristow showed that normal mitochondrial respiration suppresses tumorigenesis [98,99]. Finally, mitochondrial transplantation—in which functional mitochondria are transplanted into cancer cells—could increase cancer cell death exactly like apoptosis. Mitochondrial transfers work differently. They are a natural process of sharing mitochondria between cells, favoring cancer cells. This induces an increase in chemoresistance and the invasive nature of cancer cells that receive the mitochondria of cells from the tumor microenvironment [100].

## 5. Cancer Stem Cells (CSCs) Are Induced by Mitochondrial Damage in Stem Cells: The Origin of Tumorigenesis

**Figure 3:**
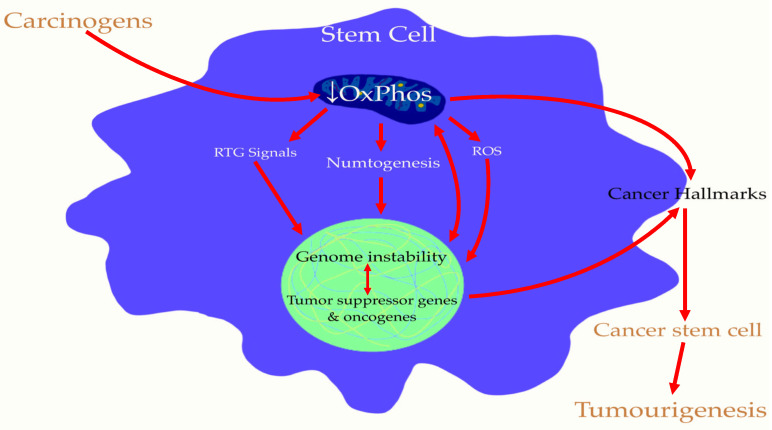
The OxPhos alteration in stem cells induces the Cancer Stem Cells. This figure represents the intracellular mechanisms necessary for the tumorigenesis. OxPhos—Oxidative Phosphorylation; ROS—Reactive Oxygen Species; RTG—ReTroGrade.

CSCs share similar properties with embryonic and adult stem cells: they self-renew in order to maintain the CSCs reservoir, as opposed to non-cancer stem cells (non-CSCs) [101]. They also have the ability of multi-potentiality, which means they can differentiate into all of the cellular components present in the tumor or organ in question, resulting in tumor heterogeneity [101]. Unlike differentiated cancer cells, CSCs are highly tumorigenic [102,103,104] and can generate a tumor identical to the original one [105]. They have been identified in all different types of cancers [101,106]. CSCs are highly involved in tumorigenesis, metastasis, treatment resistance, and tumor progression [107]. Most, if not all, cancers are believed to arise from a small number of CSCs [108]. The proportion of CSCs in tumor tissues, usually representing only around 2% of the total tumor mass, can vary significantly between different types of cancer [109,110]. Studies have shown that as few as 100 CSCs from the brain, breast, pancreas, lung, and colon can initiate tumors, whereas over 10,000 cells of other non-CSCs cannot do so [111,112,113]. Injection of 1000 prostate CSCs consistently develops tumors in immunodeficient mice, whereas injection of non-CSCs does not [114]. Mitochondria determine the function of CSCs, including ROS generation, ATP generation by OxPhos, and mitochondrial dynamics. They also determine the fate of these cells, including maintenance, differentiation, survival, proliferation, and resistance. These characteristics (metabolism, oxidative stress, and cell cycle) are altered in CSCs of all types of cancers [115,116]. CSCs alter their mitochondrial characteristics such as number, morphology, dynamic, associated signaling pathways, and turnover of damaged mitochondria to limit ROS production. CSCs can maintain their reservoir of healthy mitochondria through several processes. First, there is mitochondrial fusion, which consists of the fusion of two mitochondria together, which helps maintain mitochondrial homogeneity, support mitochondrial genome integrity, and maintains the balance between energy production and cell mass. Fusion is generally controlled by proteins (mitofusin 1 and 2 and optical atrophy protein 1). However, the findings connecting mitochondrial fusion with CSCs behavior have not yet been proven [117]. Secondly, mitochondrial fission divides a given mitochondria into two daughter mitochondria. It is achieved by the positive regulation of dynamin-related protein 1, which can be recruited into the mitochondrial membrane from the cytoplasm with the help of mitochondrial receptor proteins (mitochondrial fission 1 protein, mitochondrial dynamics proteins of 49 and 51, mitochondrial fission factor, and dynamin 2). Finally, mitophagy serves to eliminate any damaged or defective mitochondria. Mitophagy is categorized into two subtypes depending upon the protein involved, such as PTEN-induced kinase 1/Parkin-dependent mitophagy and receptor-mediated mitophagy [106]. All of these processes contribute to providing energy for better adaptation of CSCs with stress microenvironment. Age-related deterioration induces ROS accumulation, OxPhos inefficiency, and dysfunction of autophagy/mitophagy mechanisms necessary for maintaining stem cell homeostasis [118], which could increase the risk of cancer with age [118,119]. As previously stated, chemical agents are highly efficient to induce cancer in vivo [10]. In chemically induced cancers, the lesion usually occurs first in the tissue stem cells [120]. Therefore, ROS disrupts mitochondrial homeostasis and OxPhos [45], resulting in the tumorigenicity by CSCs [121]. Consequently, it is suggested that the mitochondrial–stem cell connection (MSCC) highlighted by impaired OxPhos in one or more stem cells could lead to the formation of CSCs and, thus, tumorigenesis. This is shown below in Figure 3:

To illustrate the MSCC, a community of dwarfs in Ecuador called Laron dwarfs has a very low incidence of cancer [122,123]. They have a congenital deficiency of Insulin-like Growth Factor one (IGF-1) due to a syndrome called Laron syndrome [124]. Inhibition of IGF-1 receptors has been shown to stimulate pathways that regulate mitochondrial maintenance, including mitophagy [125]. It was demonstrated in dwarf mice with Laron syndrome that they could have a higher number of bone marrow stem cells [126]. Ames dwarf mice generally live longer than other mice, and the lack of growth hormone (GH) secretion results in undetectable levels of plasma IGF-1, just like Laron dwarfs. The consequences of the absence of IGF-1 in those mice are an increase in mitochondrial respiratory activity (OxPhos), a decrease in ROS production, and a mitochondrial DNA/nuclear DNA ratio identical to other mice [127]. In contrast, the upregulation of IGF-1 receptors promotes stem cell tumorigenesis and inflammation [128]. All this seems to confirm the hypothesis of tumorigenesis due to altered OxPhos in one or more stem cells.

## 6. The Metabolism of Cancer Cells Compensates for OxPhos Insufficiency

**Figure 4:**
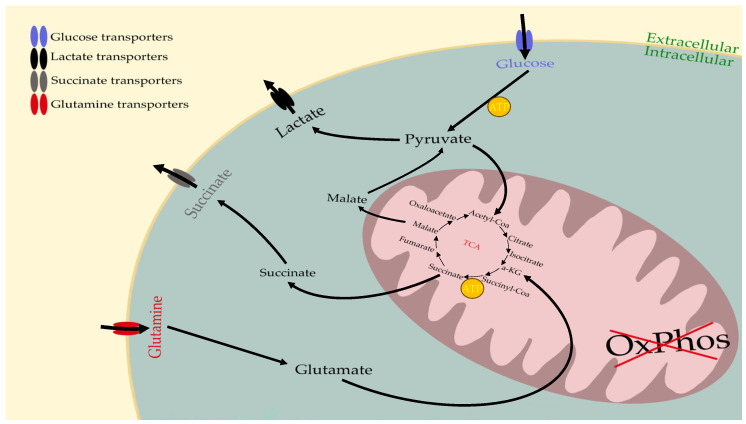
The glycolysis and glutaminolysis pathways in cancer cells. Glucose is taken up into the cytoplasm by specific glucose transporters. α-KG—α-KetoGlutarate; ATP—Adenosine Triphosphate; OxPhos—Oxidative Phosphorylation; TCA—Tricarboxylic/Citric Acid.

Cell metabolism is altered in cancer. Cancer cells can find many alternative pathways for their energy needs using glucose, glutamine, lipids, amino acids, and ketone bodies [115,129] and the CSCs have the same energy requirements as non-CSCs [130,131,132]. Healthy cells produce energy in the form of ATP mainly via two processes, the substrate-level phosphorylation and the OxPhos [133]. Under normal conditions, during the complete oxidation of glucose, 32 ATP molecules are produced through OxPhos [134]. The best-known effect is the Warburg effect: in all cancers, glucose fermentation enables cell growth since mitochondrial respiration is not sufficient. According to Warburg, glucose fermentation compensates for respiratory failure, and cancer cells continue to ferment whether oxygen is present or not [2,65,135,136]. This phenomenon (Warburg effect) is known as aerobic fermentation. The glycolytic pathway can increase ATP production 100 times faster than OxPhos can [137,138]. On the other hand, the inhibition of glycolysis leads to the death of CSCs [129]. This metabolism allows us to understand the low incidence of cancer in Laron dwarfs. Indeed, the Laron dwarfs have a normal glycemic index, hypoinsulinemia, reduced responses to glucose and insulin, and they have adequate cellular glucose uptake, which all result in the reduced potential development of the Warburg Effect in this population [139]. Another important pathway that compensates for deficiencies in OxPhos is glutaminolysis. The importance of glutamine-driven mitochondrial substrate-level phosphorylation (mSLP) in the glutaminolysis pathway is the second source of ATP for insufficient OxPhos [140]. Glutamine depletion would cause an increase in ROS and lead to a gradual decrease of CSCs [141]. The more malignant a cell becomes the greater the necessity for substrate-level phosphorylation (glycolysis, glutaminolysis) for dysregulated cell growth [142]. Therefore, the degree of malignancy can be linked directly to the energy transition from OxPhos to substrate-level phosphorylation [61]. OxPhos insufficiency leads to the up-regulation of hypoxia-inducible factor 1α (HIF-1α) and c-Myc by oncogenes, increasing glucose and glutamine transporters and thus the glycolysis and glutaminolysis pathways [72,143]. It is also due to these main fuels that it is possible to detect cancers, proving the necessity and ability of tumor cells to capture these fuels. Indeed, radioactive glucose is used as a cancer detection method [144]. Cancer cells take up radioactive glucose, allowing positron emission tomography (PET-Scan) to detect them. The same process is also possible with radioactive glutamine [145]. The glycolysis and glutaminolysis pathways are summarized below in Figure 4:

Paradoxically, the energy needs of cancer cells and healthy cells are sensibly identical. The ∆G′ of ATP (standard energy of ATP hydrolysis under physiological conditions) is relatively similar for healthy and cancerous cells. It should be minus 56 kilojoules, meaning that the efficiency of using ATP is similar in cancer cells and normal cells [61]. Indeed, cancer cells are locked into producing ATP through substrate-level phosphorylation, which is insufficient and dependent on an abundant availability of fermentable fuels like glucose and glutamine. To compensate, cancer cells can express the cytoplasmic pyruvate kinase muscle isozyme 1 and 2 (PKM1 and PKM2) isoforms at the last step of glycolysis. PKM1 produces ATP while PKM2 produces little to none [146,147]. Estimating the number of ATP molecules produced by cancer cells can be challenging. Generally, two ATP molecules are produced in the cytoplasm and two others in the mitochondria, but many cancers express the PKM2 and PKM1 isoforms, with only PKM1 generally creating ATP. Consequently, since both PKM isoforms produce lactate but only PKM1 produces ATP, lactate production cannot be used as an accurate marker for ATP synthesis through cytoplasmic glycolysis in tumor cells [147]. Persi et al. [148] showed through their analyses that the high rate of ATP production observed in cancer comes from the cytosol and not from the mitochondria. However, Persi et al. do not take into account the PKM isoforms and the synthesis of ATP by phosphorylation at the mitochondrial substrate induced by glutamine. There is still some confusion regarding CSCs and the use of OxPhos in some studies [117]. First of all, CSCs are known to be able to survive in hypoxic environments and, therefore, in the absence of OxPhos [149]. Indeed, hypoxia maintains CSCs stemness and promotes resistance through the activation of self-renewal signaling pathways [150]. The confusion may also be largely due to a tool problem. Indeed, the oxygen consumption rate (OCR) is not an accurate surrogate for ATP synthesis through OxPhos in tumor cells. The Seahorse tool is generally used as the primary tool to detect ATP from OxPhos in CSCs [115], but it can only infer that ATP flux is linked to OCR, and it is not yet able to distinguish ATP synthesis from mSLP or OxPhos [61,151]. For these reasons, in cancer cells, OxPhos is neither necessary nor sufficient for tumorigenesis [152] and for cancer progression, as tumor cells can grow in cyanide or in deep hypoxia [153,154,155,156,157]. Pastò et al. [158] also state in their paper that CSCs cannot survive in the absence of pyruvate (the glycolysis end-product) and glutamine. However, it remains unclear whether glycolysis and OxPhos (of healthy mitochondria) can coexist or are mutually exclusive [159]. Thus, as no tumor cells seem to be able to grow and survive in the absence of glucose and glutamine [3,160,161], cancer could correspond more to a metabolic pathology [12]. Glucose and glutamine-driven fermentation by glycolysis and glutaminolysis pathways are cancer cells’ metabolic signatures [151].

## 7. CSCs and Macrophages: The Origin of Metastases

CSCs induced by the MSCC are directly involved in metastases. Several authors have reported the presence of CSCs in metastases of different cancer types [162,163,164,165]. Unlike their differentiated progeny, CSCs can undergo unlimited self-renewing division [166,167] and, therefore, are able to recreate a distant tumor [168]. For metastases to form, cancer cells must be capable of intravasating into the bloodstream, surviving there, attaching, and recreating a tumor and its microenvironment. CSCs’ involvement in the metastatic process seems evident given their adaptive properties [169] partly due to their multipotent capacity [101]. They can initiate a new tumor while conserving the primary tumor’s cellular signature [170]. However, CSCs, such as CT-2A and VM-NM1 (murine glioma cell lines), cannot metastasize alone [171]. Another structure seems to have to intervene, namely the macrophages. Macrophages are commonly considered to be one of the key structures in metastases, as they have the capacity to intravasate [172] and may represent up to 50% of the tumor mass [173]. They are generally referred to as “tumor-associated macrophages” [174,175]. A phenomenon called fusion hybridization may involve macrophages to form a hybrid cell necessary for metastases. This fusion can occur with CSCs and/or differentiated macrophages [171]. Moreover, data suggests that macrophages can also allow non-CSCs to reacquire stemness properties [176], a phenomenon known as dedifferentiation [177,178]. However, CSCs may have a higher propensity to fuse with macrophages as their proximity or direct contact with intratumoral macrophages is more frequent than with non-CSCs [176]. Thus, the systemic metastases can rarely originate directly from CSCs, but most often from fusion hybridization between CSCs and macrophages [171].

## 8. The Tumor Microenvironment of CSCs: A Necessary Consequence of Mitochondrial Impairment

The tumor microenvironment plays a crucial role in the development and growth of tumors, and it is identical for cancer cells and cancer stem cells. Chronic OxPhos insufficiency affects the nucleus, in particular genomic instability. OxPhos insufficiency leads to the up-regulation of HIF-1α and the c-Myc (see Figure 1) by oncogenes, increasing glucose and glutamine transporters and thus the glycolysis and glutaminolysis pathways, which allows the development of the tumor microenvironment and their various elements. The tumor microenvironment includes pH, hypoxia, entropy, pressure and deformation, temperature, stroma, cytoplasmic water, and bioelectricity. All of these elements, essential for the maintenance and survival of cancer cells (CSCs and non-CSCs), are presented below:

### 8.1. Pressure and Deformation of Tissues

Paradoxically, tumor tissues are often detected by palpation despite cancer cells being softer than normal cells and cellular stiffness decreasing with cancer progression [179]. The rigidity may not originate from the tumor cells themselves but rather from their microenvironment. Tumors often exhibit high osmotic pressure, which is mainly caused by tumor debris and an increase in extracellular matrix components. This is a consistent characteristic of the tumor microenvironment [180]. The high osmotic pressure in tumors is consistent with the high pressure of the following fluids: interstitial fluid, blood, and lymph [181,182].

### 8.2. pH

One of the consequences of the Warburg Effect is the alteration of cellular pH. Indeed, the Warburg Effect leads to an increase in the absorption of glucose, which will be transformed into pyruvate and then into lactic acid [183]. The expression of PKM1 and PKM2 at the last step of glycolysis, observed in many tumors, contributes to the production of lactic acid [146,184]. The glutaminolysis pathway produces very little lactate [147]. The end-product of the glutaminolysis pathway is succinate (see Figure 4), which also contributes to extracellular acidification [185,186]. Furthermore, hyperosmolarity contributes to pH acidification by increasing extracellular Na+ concentration levels, which promotes aerobic glycolysis and leads to lactate accumulation [180,187,188]. Lactagenesis is a highly orchestrated effort that contributes to glycolysis, glutaminolysis, lactate production, a decrease of mitochondrial functions, angiogenesis, immunosuppression, cell migration, and metastases [185,189,190]. Lactic acid will be evacuated as waste into the extracellular environment, thus decreasing the extracellular pH [191]. In normal cells, the intracellular pH typically ranges from 6.9 to 7.2, while the extracellular pH ranges from 7.2 to 7.4. Nuclear magnetic resonance imaging studies have shown a range between 7.2 and 7.7 of intracellular tumor pH, while the extracellular tumor pH ranges between 6.2 and 6.8 [192,193,194]. The high production of protons and their migration to the extracellular environment are responsible for establishing this pH gradient in cancer cells [195]. Therefore, the genomic instability and somatic mutations observed in most cancers are a result of the chronic production of ROS (caused by the alteration of OxPhos) and microenvironment acidification [76]. Solid and liquid tumors seem to have the same fuels, glucose, and glutamine [186,196,197,198,199], and, therefore, they have the same acidic environment. Indeed, a dependency on glucose and glutamine fermentation leads to the extracellular accumulation of lactic acid and succinic acid, which will acidify the microenvironment, leading to tumor progression.

### 8.3. Hypoxia

Hypoxia is another major component of the tumor environment. Mitochondria-derived ROS can promote cancer initiation through oxidative stress and cancer cell progression via hypoxia-inducing factors, particularly HIF-1α [54]. The activation of RTGs leads to the persistent expression of various oncogenes such as HIF-1α, which induces hypoxia and positively regulates the glycolysis and glutaminolysis pathways (and indirectly the pH) [142]. HIF is also expressed when ROS production increases [200]. Tumor cells are exposed to a continuum of oxygen concentration. The larger the tumor is, the farther some cancer cells will be from the large vessels, resulting in these distant cells being found in a hypoxic environment [201,202]. The more hypoxic a cell is, the less access it has to OxPhos, leading the cell to rely more on substrate-level phosphorylation as an energy source [61,203]. Hypoxia also contributes to the alteration of anti-tumor immune responses [204].

### 8.4. Entropy

Entropy represents the difficulties of waste evacuation. Cells typically generate heat through the mitochondria and ATP, and this process is disrupted in cancer cells. This leads to increased waste and a disorder called entropy, which is necessary for the cellular heterogeneity of cancer [205]. The loss of energy (ATP) has an immediate influence on the transport of matter and on the preservation of cellular information, leading to disturbance and, therefore, an increase in entropy [206].

### 8.5. Temperature

One study has shown that the temperature in tumor environments is generally around 1 °C higher than in healthy environments [207]. Moreover, mitochondrial temperature is higher in cancer cells compared to normal cells [208].

### 8.6. Stroma

The stroma includes the extracellular matrix, fibrous proteins such as collagen, growth factors, antibodies, metabolites, mesenchymal support cells, blood vessels, lymphatic vessels, nerves, and immune system cells [209]. In some instances, the alteration in mtDNA can activate mitochondrial retrograde pathways. These pathways lead to epithelial-to-mesenchymal transition-like reprogramming that can promote tumorigenesis and migration. Moreover, mitochondrial RTG signals could regulate Wnt signaling, which can promote tumorigenesis [117]. Stroma has a significant impact on the formation of the tumor microenvironment [210].

### 8.7. Bioelectricity and Cancer

Cancer is characterized by an excessive accumulation of electrons, which reduces the cells’ electrical conduction. Higher rates of glycolysis lead to increased electron transfer [211], which is a major contributing factor to the increased production of ROS [212]. In eukaryotic cells, microtubules generating the electromagnetic field comprise tubulin heterodimers with a strong electric dipole. The Warburg Effect in cancer cells results in a reduction in electromagnetic field strength, disrupted coherence, and an increase in oscillation frequency [213]. A comparison of the space between the internal membrane and the mitochondrial matrix of healthy and cancer cells reveals a reduction in the proton gradient, an establishment of the ordered water layer, an increase in electron emissions, and consequently, damping of the electromagnetic field in cancer cells [214]. Behnam et al. [215] suggested targeting the mitochondria with magnetic fields in order to restore the initial mitochondrial magnetic field by reducing the quantity of electrons.

### 8.8. Cytoplasmic Water

In a healthy environment, water molecules carry out three types of motion in space known as vibration, self-rotation, and translation [216]. In cancer, the dynamics of hydration water molecules remain virtually unchanged during the transition from healthy to cancer cells. However, the rotational movements of cytoplasmic water undergo significant changes during the transition from healthy to cancer cells [217]. Tanner et al. [218] observed alterations and loss of rotation in malignant cells as opposed to healthy cells. It is possible that the rotation of cytoplasmic water molecules is altered due to the loss of the mitochondrial magnetic field.

**Figure 5:**
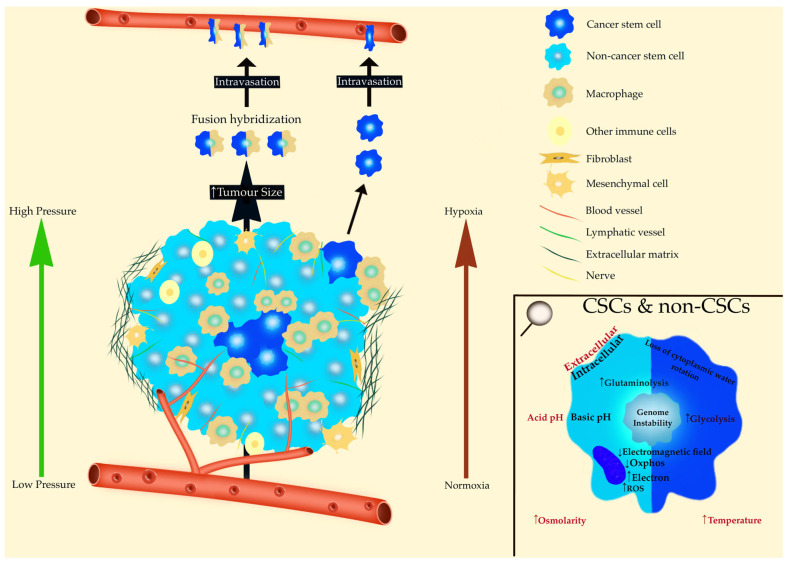
The tumor-scale microenvironment is shown on the left. In the enlarged window at the bottom right, the microenvironment is represented at the cellular level. This environment is identical for cancer cells and cancer stem cells. CSCs—Cancer Stem Cells; non-CSCs—non-Cancer Stem Cells; OxPhos—Oxidative Phosphorylation; ROS—Reactive Oxygen Species.

All of these phenomena are summarized below in Figure 5:

The microenvironment has a major role in all stages of cancer, from initiation to metastases [219,220], and mitochondria can regulate and modify the microenvironment [221].

## 9. The CSCs (and Their Mitochondria), an Explanation of the Current Treatments and Relapses

In light of the MSCC, restoring the OxPhos metabolism of CSCs represents a relevant therapeutic lead for potential cancer management [109,222,223,224], perhaps in combination with therapies that also target macrophages [225]. Indeed, most current cancer drugs target tumor growth by inhibiting DNA synthesis or cell division of actively dividing cancer cells [226,227,228], but CSCs are often in a quiescent state [229]. The quiescent state explains the slow growth of cancers [230]. Indeed, chemoradiotherapy targets bulk cancer cells, leading to their death, while CSCs are free to grow and improve their resistance [106,231,232,233,234]. CSCs can continue to proliferate as they lack the mutation of the oncogene “targeted” by the drug and, therefore, do not respond to treatment [235,236]. This may explain the slight improvement with new therapies. Ladanie et al. showed that over the past fifteen years, the improvement in overall survival by new therapies is 2.4 months [237]. Another study reports an improvement of 3.4 months over the last thirty years [238]. Additionally, current therapies do not restore OxPhos and can sometimes alter it [44,223,239,240,241,242]. The lack of OxPhos restoration allows a better understanding of why many patients only have a partial response and end up relapsing, showing a tumor that is often more resistant and more conducive to the formation of metastases [243,244]. Indeed, chemoradiotherapy can induce prometastatic niches in other organs [245], in which macrophages will then frequently fuse with CSCs to reinforce resistance to treatments [174]. Moreover, the majority of detected cancers are non-metastatic and typically require surgery in combination with standard multitherapy [246]. A significant proportion of healing can be attributed to the combination of existing treatments that target the bulk of cancer cells [231] and surgeries that remove CSCs through excision. Indeed, melanomas, thyroid cancers, kidney cancers, prostate cancers, uterine corpus cancers, testicular cancers, and breast cancers are excellent examples of the effectiveness of surgery on non-metastatic cancers [247]. Another example is pancreatic cancer, which has the lowest survival rate among all cancers. When the tumor is resectable (rare), excision is the only possible cure for this cancer [248]. The primary challenge in cancer treatment remains metastases [249], which account for approximately 90% of cancer deaths [249] and are rarely operable [247]. Standard therapies do not eliminate CSCs (and their mitochondria) [231,239,240,250], which, associated with macrophages, can form metastases [171]. The relapse phenomenon follows the same logic. Considering that CSCs cannot be targeted and represent ≈ 2% of the total tumor mass [109,110,251,252], the vast majority of tumor-forming cells (bulk cancer cells, macrophages, etc.) can die after conventional therapies [231,253,254], allowing the reduction or even temporary disappearance of the tumor [255]. Yet, the new PET-Scan models have a detection limit of 4 mm [256], knowing that a 5 mm cancer has approximately 10 million cells and a 1 mm cancer has approximately 100,000 cells [257]. Since the lower detection limit of a PET-Scan is around 10^5^ to 10^6^ malignant cells [258], CSCs can fall below the PET-Scan detection threshold, giving the impression of remission before reappearing a few months or years later. Many agents can target the pathways that are associated with CSCs, such as Vismodegib, Glasdegib, MK-0752, OMP-54F28, and Selinexor [259]. However, these agents do not specifically target the mitochondria and, more particularly, do not induce the restoration of OxPhos. Many other therapeutic strategies have been proposed to target mitochondria, such as Resveratrol, Metformin, and Melatonin. These can target the mitochondrial dynamics or OxPhos [260]. For example, drugs such as Metformin or Phenformin can alter OxPhos by electron transport chain (ETC) inhibitors. Antibiotics such as Doxycycline, Tigecycline, and Bedaquiline can target mitochondrial biogenesis. The drug Mdivi-1 targets mitochondrial dynamics, while 188Re-liposome and the inhibitor liensinine block the mitophagy [115,117]. Most of the time, these treatments do not restore mitochondrial homeostasis [130], but mainly restore or alter only a few parts of the dysfunction. This seems to be insufficient. In addition, therapies that might involve targeting mitochondria in tumor cells have to be used cautiously. Mitochondrial targeting can have lethal off-target effects on OxPhos metabolism in normal host cells. These toxic effects include nausea, seizures, and even coma [261,262]. Therapies aimed at increasing OxPhos, such as hyperbaric oxygen therapy, could be relevant. This would increase the activity of OxPhos and would have tumor suppressor effects [263]. A ketogenic diet or fasting could inhibit the fuels necessary for cancer cells while increasing the activity of OxPhos as well [264].

## 10. Key Points of the MSCC

**Figure 6:**
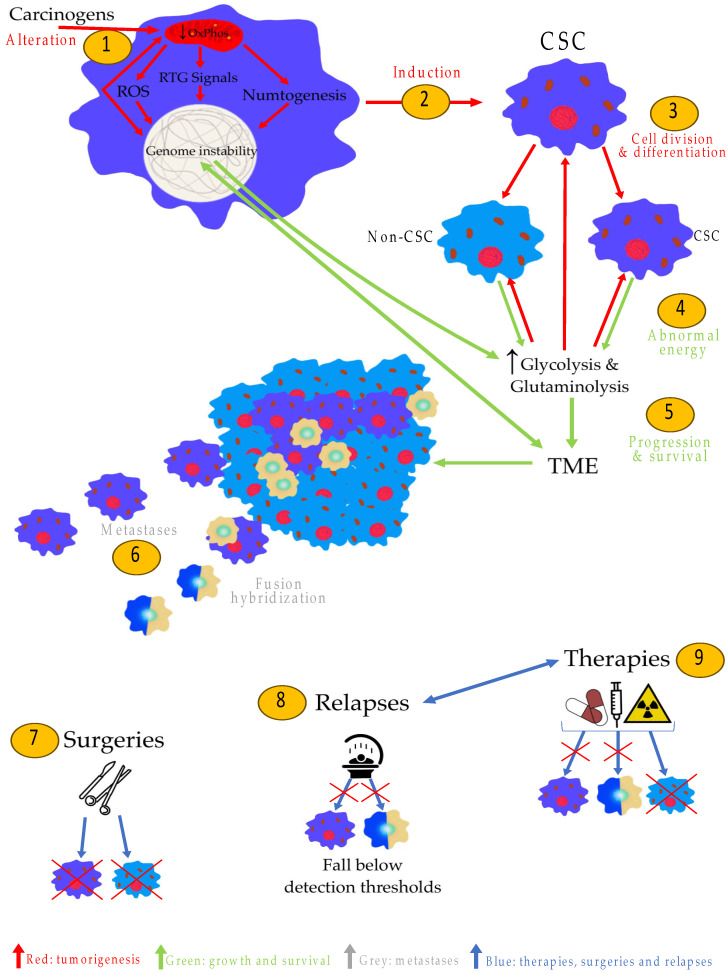
Synthesis of MSCC. (1) Carcinogens alter OxPhos in stem cell(s). (2) OxPhos alteration in stem cells induces cancer stem cells (CSCs). (3) CSCs can be divided into other CSCs or non-cancer stem cells (non-CSCs). (4) Metabolism of cancer cells compensates for OxPhos insufficiency with glycolysis and glutaminolysis. The chronic ROS, the RTG signals, and the numtogenesis induce overexpression of oncogenes and inactivation of tumor-suppressor genes, which contribute to abnormal energy metabolism in cancer. (5) The abnormal metabolism and oncogenes contribute to generating the tumor microenvironment (TME). The acidification, inflammation, and chronic production of ROS contribute to genome instability and somatic mutations. (6) Metastases are produced by CSCs directly (rare) or by fusion hybridization with macrophages. (7) Local surgeries in non-metastatic cancers can target non-CSCs and CSCs. (8) CSCs and their fusion hybridizations with macrophages are present in low numbers and can fall below detection thresholds. (9) Therapies can target only non-CSCs. CSCs—Cancer Stem Cells; non-CSCs—non-Cancer Stem Cells; OxPhos—Oxidative Phosphorylation; ROS—Reactive Oxygen Species; RTG—ReTroGrade; TME—Tumor Microenvironment.

Chronic insufficiency of OxPhos in a stem cell may be a keystone of cancer and its different steps, according to MSCC. The approach provided by the MSCC is summarized in Figure 6.

(1) Tumorigenesis: OxPhos alteration in one or more stem cells (MSCC) creates the CSCs, which could lead to tumorigenesis.

(2) Growth and survival: OxPhos insufficiency induces genomic instability and alters the expression of oncogenes and tumor-suppressor genes, which can contribute to the tumor microenvironment and abnormal energy metabolism required for tumor survival and progression. Cancer cells (CSCs and non-CSCs) depend critically on fermentable fuels, namely glucose and glutamine.

(3) Metastases: The metastases can rarely originate from either CSCs (induced by MSCC) directly but more frequently from fusion hybridization between CSCs and macrophages. The tumor progression and malignancy can be directly linked to the energy transition from OxPhos to substrate-level phosphorylation (glycolysis, glutaminolysis). Thus, the higher the degree of mitochondrial abnormalities (decrease in OxPhos), the greater the degree of malignancy.

(4) Healings, failures, and relapses: Curing cases can be explained by the capacity of standard care to target the CSCs, while treatment failures may be attributed to their inability to do so. Relapses may occur due to the low number of CSCs (alone or fused with macrophages), which could fall below detection thresholds despite their presence.

## 11. Conclusions

According to the MSCC approach, tumorigenesis could result from a mitochondrial alteration (metabolic model) in one or several stem cells (CSCs models) forming the CSCs. Genomic instability and somatic mutations are effects of insufficient OxPhos leading to abnormal energy metabolism and a tumor microenvironment (TOFT), contributing to cancer cell progression and survival. The reviewed information shows that the respiratory impairment of stem cells could be one keystone of cancer and is necessary for all the different steps, from tumorigenesis to metastases. The metabolism also plays a significant role in cancer, since the tumor must compensate for the altered OxPhos, mostly through glycolysis and glutaminolysis. This MSCC approach opens up many therapeutic leads for cancer management. The first would involve the simultaneous restricting of glucose and glutamine availability while transitioning the body to non-fermentable fuels like ketone bodies and fatty acids. The second approach would be specifically restoring cellular respiration (OxPhos) in CSCs. An important goal in preclinical models and human cancer patients will be to demonstrate that the restoration of OxPhos, combined with simultaneous targeting of glucose and glutamine while under therapeutic ketosis, should permit the elimination of CSCs and thus reduce tumorigenesis and metastases.

## Data Availability

Not applicable.

## References

[1] Majérus M.-A. (2022). The cause of cancer: The unifying theory. Adv. Cancer Biol.-Metastasis.

[2] Warburg O. (1956). On the origin of cancer cells. Science.

[3] Seyfried T.N., Chinopoulos C. (2021). Can the Mitochondrial Metabolic Theory Explain Better the Origin and Management of Cancer than Can the Somatic Mutation Theory?. Metabolites.

[4] Hanahan D., Weinberg R.A. (2000). The hallmarks of cancer. Cell.

[5] Capp J.P. (2019). Cancer Stem Cells: From Historical Roots to a New Perspective. J. Oncol..

[6] Soto A.M., Sonnenschein C. (2011). The tissue organization field theory of cancer: A testable replacement for the somatic mutation theory. Bioessays.

[7] Anand P., Kunnumakkara A.B., Sundaram C., Harikumar K.B., Tharakan S.T., Lai O.S., Sung B., Aggarwal B.B. (2008). Cancer is a preventable disease that requires major lifestyle changes. Pharm. Res..

[8] Klement R.J. (2024). Cancer as a global health crisis with deep evolutionary roots. Glob. Transit..

[9] Onaciu A., Munteanu R., Munteanu V.C., Gulei D., Raduly L., Feder R.I., Pirlog R., Atanasov A.G., Korban S.S., Irimie A. (2020). Spontaneous and Induced Animal Models for Cancer Research. Diagnostics.

[10] Zhou Y., Xia J., Xu S., She T., Zhang Y., Sun Y., Wen M., Jiang T., Xiong Y., Lei J. (2023). Experimental mouse models for translational human cancer research. Front. Immunol..

[11] Adjiri A. (2017). DNA Mutations May Not Be the Cause of Cancer. Oncol. Ther..

[12] Cao S., Zhang C., Xu Y. (2015). Somatic mutations may not be the primary drivers of cancer formation. Int. J. Cancer.

[13] Woodman S.E., Mills G.B. (2010). Are oncogenes sufficient to cause human cancer?. Proc. Natl. Acad. Sci. USA.

[14] Brash D., Cairns J. (2009). The mysterious steps in carcinogenesis. Br. J. Cancer.

[15] Balmain A., Harris C.C. (2000). Carcinogenesis in mouse and human cells: Parallels and paradoxes. Carcinogenesis.

[16] Morales C.P., Holt S.E., Ouellette M., Kaur K.J., Yan Y., Wilson K.S., White M.A., Wright W.E., Shay J.W. (1999). Absence of cancer-associated changes in human fibroblasts immortalized with telomerase. Nat. Genet..

[17] Plattner R., Anderson M.J., Sato K.Y., Fasching C.L., Der C.J., Stanbridge E.J. (1996). Loss of oncogenic ras expression does not correlate with loss of tumorigenicity in human cells. Proc. Natl. Acad. Sci. USA.

[18] de Magalhães J.P. (2022). Every gene can (and possibly will) be associated with cancer. Trends Genet..

[19] Wood L.D., Parsons D.W., Jones S., Lin J., Sjöblom T., Leary R.J., Shen D., Boca S.M., Barber T., Ptak J. (2007). The genomic landscapes of human breast and colorectal cancers. Science.

[20] Dentro S.C., Leshchiner I., Haase K., Tarabichi M., Wintersinger J., Deshwar A.G., Yu K., Rubanova Y., Macintyre G., Demeulemeester J. (2021). Characterizing genetic intra-tumor heterogeneity across 2658 human cancer genomes. Cell.

[21] Lawrence M.S., Stojanov P., Polak P., Kryukov G.V., Cibulskis K., Sivachenko A., Carter S.L., Stewart C., Mermel C.H., Roberts S.A. (2013). Mutational heterogeneity in cancer and the search for new cancer-associated genes. Nature.

[22] Martincorena I., Fowler J.C., Wabik A., Lawson A.R.J., Abascal F., Hall M.W.J., Cagan A., Murai K., Mahbubani K., Stratton M.R. (2018). Somatic mutant clones colonize the human esophagus with age. Science.

[23] Yokoyama A., Kakiuchi N., Yoshizato T., Nannya Y., Suzuki H., Takeuchi Y., Shiozawa Y., Sato Y., Aoki K., Kim S.K. (2019). Age-related remodelling of oesophageal epithelia by mutated cancer drivers. Nature.

[24] Yizhak K., Aguet F., Kim J., Hess J.M., Kübler K., Grimsby J., Frazer R., Zhang H., Haradhvala N.J., Rosebrock D. (2019). RNA sequence analysis reveals macroscopic somatic clonal expansion across normal tissues. Science.

[25] Li C., Williams S.M. (2013). Human Somatic Variation: It’s Not Just for Cancer Anymore. Curr. Genet. Med. Rep..

[26] Zhang L., Zhou W., Velculescu V.E., Kern S.E., Hruban R.H., Hamilton S.R., Vogelstein B., Kinzler K.W. (1997). Gene expression profiles in normal and cancer cells. Science.

[27] Martincorena I., Campbell P.J. (2015). Somatic mutation in cancer and normal cells. Science.

[28] Chanock S.J. (2018). The paradox of mutations and cancer. Science.

[29] Gormally E., Vineis P., Matullo G., Veglia F., Caboux E., Le Roux E., Peluso M., Garte S., Guarrera S., Munnia A. (2006). TP53 and KRAS2 mutations in plasma DNA of healthy subjects and subsequent cancer occurrence: A prospective study. Cancer Res..

[30] Krimmel J.D., Schmitt M.W., Harrell M.I., Agnew K.J., Kennedy S.R., Emond M.J., Loeb L.A., Swisher E.M., Risques R.A. (2016). Ultra-deep sequencing detects ovarian cancer cells in peritoneal fluid and reveals somatic TP53 mutations in noncancerous tissues. Proc. Natl. Acad. Sci. USA.

[31] Rème T., Travaglio A., Gueydon E., Adla L., Jorgensen C., Sany J. (1998). Mutations of the p53 tumour suppressor gene in erosive rheumatoid synovial tissue. Clin. Exp. Immunol..

[32] Yamanishi Y., Boyle D.L., Rosengren S., Green D.R., Zvaifler N.J., Firestein G.S. (2002). Regional analysis of p53 mutations in rheumatoid arthritis synovium. Proc. Natl. Acad. Sci. USA.

[33] Firestein G.S., Echeverri F., Yeo M., Zvaifler N.J., Green D.R. (1997). Somatic mutations in the p53 tumor suppressor gene in rheumatoid arthritis synovium. Proc. Natl. Acad. Sci. USA.

[34] Mendiratta G., Ke E., Aziz M., Liarakos D., Tong M., Stites E.C. (2021). Cancer gene mutation frequencies for the U.S. population. Nat. Commun..

[35] Soto A.M., Sonnenschein C. (2004). The somatic mutation theory of cancer: Growing problems with the paradigm?. Bioessays.

[36] Hernández L.G., van Steeg H., Luijten M., van Benthem J. (2009). Mechanisms of non-genotoxic carcinogens and importance of a weight of evidence approach. Mutat. Res..

[37] Lijinsky W. (1990). Non-genotoxic environmental carcinogens. Environ. Carcinog. Rev..

[38] Bischoff F., Bryson G. (1964). Carcinogenesis through solid state surfaces. Prog. Exp. Tumor. Res..

[39] Mally A., Chipman J.K. (2002). Non-genotoxic carcinogens: Early effects on gap junctions, cell proliferation and apoptosis in the rat. Toxicology.

[40] Monti N., Verna R., Piombarolo A., Querqui A., Bizzarri M., Fedeli V. (2022). Paradoxical Behavior of Oncogenes Undermines the Somatic Mutation Theory. Biomolecules.

[41] Brücher B.L.D.M., Jamall I.S. (2016). Somatic Mutation Theory—Why it’s Wrong for Most Cancers. Cell. Physiol. Biochem..

[42] Vyas S., Zaganjor E., Haigis M.C. (2016). Mitochondria and Cancer. Cell.

[43] O’Malley J., Kumar R., Inigo J., Yadava N., Chandra D. (2020). Mitochondrial Stress Response and Cancer. Trends Cancer.

[44] Kuretu A., Arineitwe C., Mothibe M., Ngubane P., Khathi A., Sibiya N. (2023). Drug-induced mitochondrial toxicity: Risks of developing glucose handling impairments. Front. Endocrinol..

[45] Hoogstraten C.A., Lyon J.J., Smeitink J.A.M., Russel F.G.M., Schirris T.J.J. (2023). Time to Change: A Systems Pharmacology Approach to Disentangle Mechanisms of Drug-Induced Mitochondrial Toxicity. Pharmacol. Rev..

[46] Reddam A., McLarnan S., Kupsco A. (2022). Environmental Chemical Exposures and Mitochondrial Dysfunction: A Review of Recent Literature. Curr. Environ. Health Rep..

[47] Kam W.W., Banati R.B. (2013). Effects of ionizing radiation on mitochondria. Free Radic. Biol. Med..

[48] Elesela S., Lukacs N.W. (2021). Role of Mitochondria in Viral Infections. Life.

[49] El Sayed S.M. (2023). Biochemical Origin of the Warburg Effect in Light of 15 Years of Research Experience: A Novel Evidence-Based View (An Expert Opinion Article). OncoTargets Ther..

[50] Iranmanesh Y., Jiang B., Favour O.C., Dou Z., Wu J., Li J., Sun C. (2021). Mitochondria’s Role in the Maintenance of Cancer Stem Cells in Glioblastoma. Front. Oncol..

[51] Modica-Napolitano J.S., Kulawiec M., Singh K.K. (2007). Mitochondria and human cancer. Curr. Mol. Med..

[52] Yuan Y., Ju Y.S., Kim Y., Li J., Wang Y., Yoon C.J., Yang Y., Martincorena I., Creighton C.J., Weinstein J.N. (2020). Comprehensive molecular characterization of mitochondrial genomes in human cancers. Nat. Genet..

[53] Kim M., Mahmood M., Reznik E., Gammage P.A. (2022). Mitochondrial DNA is a major source of driver mutations in cancer. Trends Cancer.

[54] Sabharwal S.S., Schumacker P.T. (2014). Mitochondrial ROS in cancer: Initiators, amplifiers or an Achilles’ heel?. Nat. Rev. Cancer.

[55] Kiebish M.A., Seyfried T.N. (2005). Absence of pathogenic mitochondrial DNA mutations in mouse brain tumors. BMC Cancer.

[56] Cruz-Bermúdez A., Vallejo C.G., Vicente-Blanco R.J., Gallardo M.E., Fernández-Moreno M., Quintanilla M., Garesse R. (2015). Enhanced tumorigenicity by mitochondrial DNA mild mutations. Oncotarget.

[57] Pedersen P.L. (1978). Tumor mitochondria and the bioenergetics of cancer cells. Prog. Exp. Tumor. Res..

[58] Elliott R.L., Jiang X.P., Head J.F. (2012). Mitochondria organelle transplantation: Introduction of normal epithelial mitochondria into human cancer cells inhibits proliferation and increases drug sensitivity. Breast Cancer Res. Treat..

[59] Arismendi-Morillo G. (2009). Electron microscopy morphology of the mitochondrial network in human cancer. Int. J. Biochem. Cell Biol..

[60] Deighton R.F., Le Bihan T., Martin S.F., Gerth A.M.J., McCulloch M., Edgar J.M., Kerr L.E., Whittle I.R., McCulloch J. (2014). Interactions among mitochondrial proteins altered in glioblastoma. J. Neurooncol..

[61] Seyfried T.N., Arismendi-Morillo G., Mukherjee P., Chinopoulos C. (2020). On the Origin of ATP Synthesis in Cancer. iScience.

[62] Badrinath N., Yoo S.Y. (2018). Mitochondria in cancer: In the aspects of tumorigenesis and targeted therapy. Carcinogenesis.

[63] Nakamura H., Takada K. (2021). Reactive oxygen species in cancer: Current findings and future directions. Cancer Sci..

[64] Kudryavtseva A.V., Krasnov G.S., Dmitriev A.A., Alekseev B.Y., Kardymon O.L., Sadritdinova A.F., Fedorova M.S., Pokrovsky A.V., Melnikova N.V., Kaprin A.D. (2016). Mitochondrial dysfunction and oxidative stress in aging and cancer. Oncotarget.

[65] Warburg O. (1956). On respiratory impairment in cancer cells. Science.

[66] Buss L.G., Rheinheimer B.A., Limesand K.H. (2024). Radiation-induced changes in energy metabolism result in mitochondrial dysfunction in salivary glands. Sci. Rep..

[67] Keller B.J., Marsman D.S., Popp J.A., Thurman R.G. (1992). Several nongenotoxic carcinogens uncouple mitochondrial oxidative phosphorylation. Biochim. Biophys. Acta (BBA)—Bioenerg..

[68] Ito Y., Nakajima K., Masubuchi Y., Kikuchi S., Saito F., Akahori Y., Jin M., Yoshida T., Shibutani M. (2019). Differential responses on energy metabolic pathway reprogramming between genotoxic and non-genotoxic hepatocarcinogens in rat liver cells. J. Toxicol. Pathol..

[69] Roskelley R.C., Mayer N., Horwitt B.N., Salter W.T. (1943). Studies in cancer. VII. Enzyme deficiency in human and experimental cancer. J. Clin. Investig..

[70] Jazwinski S.M. (2014). The retrograde response: A conserved compensatory reaction to damage from within and from without. Prog. Mol. Biol. Transl. Sci..

[71] da Cunha F.M., Torelli N.Q., Kowaltowski A.J. (2015). Mitochondrial Retrograde Signaling: Triggers, Pathways, and Outcomes. Oxid. Med. Cell. Longev..

[72] Yang D., Kim J. (2019). Mitochondrial Retrograde Signalling and Metabolic Alterations in the Tumour Microenvironment. Cells.

[73] Singh K.K., Choudhury A.R., Tiwari H.K. (2017). Numtogenesis as a mechanism for development of cancer. Semin. Cancer Biol..

[74] Puertas M.J., González-Sánchez M. (2020). Insertions of mitochondrial DNA into the nucleus—Effects and role in cell evolution. Genome.

[75] Bonora M., Missiroli S., Perrone M., Fiorica F., Pinton P., Giorgi C. (2021). Mitochondrial Control of Genomic Instability in Cancer. Cancers.

[76] Bartesaghi S., Graziano V., Galavotti S., Henriquez N.V., Betts J., Saxena J., Minieri V., Deli A., Karlsson A., Martins L.M. (2015). Inhibition of oxidative metabolism leads to p53 genetic inactivation and transformation in neural stem cells. Proc. Natl. Acad. Sci. USA.

[77] Chandra D., Singh K.K. (2011). Genetic insights into OXPHOS defect and its role in cancer. Biochim. Biophys. Acta (BBA)—Bioenerg..

[78] Koura M., Isaka H., Yoshida M.C., Tosu M., Sekiguchi T. (1982). Suppression of tumorigenicity in interspecific reconstituted cells and cybrids. Gan.

[79] Israel B.A., Schaeffer W.I. (1987). Cytoplasmic suppression of malignancy. Vitro Cell. Dev. Biol..

[80] Shay J.W., Werbin H. (1988). Cytoplasmic suppression of tumorigenicity in reconstructed mouse cells. Cancer Res..

[81] Howell A.N., Sager R. (1978). Tumorigenicity and its suppression in cybrids of mouse and Chinese hamster cell lines. Proc. Natl. Acad. Sci. USA.

[82] Jonasson J., Harris H. (1977). The analysis of malignancy by cell fusion. VIII. Evidence for the intervention of an extra-chromosomal element. J. Cell Sci..

[83] McKinnell R.G., Deggins B.A., Labat D.D. (1969). Transplantation of pluripotential nuclei from triploid frog tumors. Science.

[84] Mintz B., Illmensee K. (1975). Normal genetically mosaic mice produced from malignant teratocarcinoma cells. Proc. Natl. Acad. Sci. USA.

[85] Li L., Connelly M.C., Wetmore C., Curran T., Morgan J.I. (2003). Mouse embryos cloned from brain tumors. Cancer Res..

[86] Hochedlinger K., Blelloch R., Brennan C., Yamada Y., Kim M., Chin L., Jaenisch R. (2004). Reprogramming of a melanoma genome by nuclear transplantation. Genes Dev..

[87] Maffini M.V., Calabro J.M., Soto A.M., Sonnenschein C. (2005). Stromal regulation of neoplastic development: Age-dependent normalization of neoplastic mammary cells by mammary stroma. Am. J. Pathol..

[88] Coleman W.B., Wennerberg A.E., Smith G.J., Grisham J.W. (1993). Regulation of the differentiation of diploid and some aneuploid rat liver epithelial (stemlike) cells by the hepatic microenvironment. Am. J. Pathol..

[89] McCullough K.D., Coleman W.B., Smith G.J., Grisham J.W. (1997). Age-dependent induction of hepatic tumor regression by the tissue microenvironment after transplantation of neoplastically transformed rat liver epithelial cells into the liver. Cancer Res..

[90] Kaipparettu B.A., Ma Y., Park J.H., Lee T.L., Zhang Y., Yotnda P., Creighton C.J., Chan W.Y., Wong L.J. (2013). Crosstalk from non-cancerous mitochondria can inhibit tumor properties of metastatic cells by suppressing oncogenic pathways. PLoS ONE.

[91] Israel B.A., Schaeffer W.I. (1988). Cytoplasmic mediation of malignancy. Vitro Cell. Dev. Biol..

[92] Petros J.A., Baumann A.K., Ruiz-Pesini E., Amin M.B., Sun C.Q., Hall J., Lim S., Issa M.M., Flanders W.D., Hosseini S.H. (2005). mtDNA mutations increase tumorigenicity in prostate cancer. Proc. Natl. Acad. Sci. USA.

[93] Darlington C.D. (1948). The plasmagene theory of the origin of cancer. Br. J. Cancer.

[94] Chang J.C., Chang H.S., Wu Y.C., Cheng W.L., Lin T.T., Chang H.J., Kuo S.J., Chen S.T., Liu C.S. (2019). Mitochondrial transplantation regulates antitumour activity, chemoresistance and mitochondrial dynamics in breast cancer. J. Exp. Clin. Cancer Res..

[95] Fu A., Hou Y., Yu Z., Zhao Z., Liu Z. (2019). Healthy mitochondria inhibit the metastatic melanoma in lungs. Int. J. Biol. Sci..

[96] Ma Y., Bai R.K., Trieu R., Wong L.J. (2010). Mitochondrial dysfunction in human breast cancer cells and their transmitochondrial cybrids. Biochim. Biophys. Acta (BBA)—Bioenerg..

[97] Sun C., Liu X., Wang B., Wang Z., Liu Y., Di C., Si J., Li H., Wu Q., Xu D. (2019). Endocytosis-mediated mitochondrial transplantation: Transferring normal human astrocytic mitochondria into glioma cells rescues aerobic respiration and enhances radiosensitivity. Theranostics.

[98] Ristow M. (2006). Oxidative metabolism in cancer growth. Curr. Opin. Clin. Nutr. Metab. Care.

[99] Cuezva J.M., Ortega A.D., Willers I., Sánchez-Cenizo L., Aldea M., Sánchez-Aragó M. (2009). The tumor suppressor function of mitochondria: Translation into the clinics. Biochim. Biophys. Acta (BBA)—Bioenerg..

[100] Cruz-Gregorio A., Aranda-Rivera A.K., Amador-Martinez I., Maycotte P. (2023). Mitochondrial transplantation strategies in multifaceted induction of cancer cell death. Life Sci..

[101] Ginestier C. (2017). Les cellules souches cancéreuse: Définition et techniques d’isolement. Bull. Cancer..

[102] Adams J.M., Strasser A. (2008). Is tumor growth sustained by rare cancer stem cells or dominant clones?. Cancer Res..

[103] Tamura K., Aoyagi M., Ando N., Ogishima T., Wakimoto H., Yamamoto M., Ohno K. (2013). Expansion of CD133-positive glioma cells in recurrent de novo glioblastomas after radiotherapy and chemotherapy. J. Neurosurg..

[104] Huels D.J., Sansom O.J. (2015). Stem vs non-stem cell origin of colorectal cancer. Br. J. Cancer.

[105] Kobayashi N.C., Noronha S.M. (2015). Cancer stem cells: A new approach to tumor development. Rev. Assoc. Med. Bras..

[106] Garimella S.V., Gampa S.C., Chaturvedi P. (2023). Mitochondria in Cancer Stem Cells: From an Innocent Bystander to a Central Player in Therapy Resistance. Stem Cells Cloning.

[107] Huang T., Song X., Xu D., Tiek D., Goenka A., Wu B., Sastry N., Hu B., Cheng S.Y. (2020). Stem cell programs in cancer initiation, progression, and therapy resistance. Theranostics.

[108] Keith B., Simon M.C. (2007). Hypoxia-inducible factors, stem cells, and cancer. Cell.

[109] Yang L., Shi P., Zhao G., Xu J., Peng W., Zhang J., Zhang G., Wang X., Dong Z., Chen F. (2020). Targeting cancer stem cell pathways for cancer therapy. Signal Transduct. Target. Ther..

[110] Toledo-Guzmán M.E., Bigoni-Ordóñez G.D., Ibáñez Hernández M., Ortiz-Sánchez E. (2018). Cancer stem cell impact on clinical oncology. World J. Stem Cells.

[111] Singh S.K., Hawkins C., Clarke I.D., Squire J.A., Bayani J., Hide T., Henkelman R.M., Cusimano M.D., Dirks P.B. (2004). Identification of human brain tumour initiating cells. Nature.

[112] Li C., Heidt D.G., Dalerba P., Burant C.F., Zhang L., Adsay V., Wicha M., Clarke M.F., Simeone D.M. (2007). Identification of pancreatic cancer stem cells. Cancer Res..

[113] Al-Hajj M., Wicha M.S., Benito-Hernandez A., Morrison S.J., Clarke M.F. (2003). Prospective identification of tumorigenic breast cancer cells. Proc. Natl. Acad. Sci. USA.

[114] Hurt E.M., Kawasaki B.T., Klarmann G.J., Thomas S.B., Farrar W.L. (2008). CD44+ CD24(−) prostate cells are early cancer progenitor/stem cells that provide a model for patients with poor prognosis. Br. J. Cancer.

[115] Jagust P., de Luxán-Delgado B., Parejo-Alonso B., Sancho P. (2019). Metabolism-Based Therapeutic Strategies Targeting Cancer Stem Cells. Front. Pharmacol..

[116] Chen C.L., Uthaya Kumar D.B., Punj V., Xu J., Sher L., Tahara S.M., Hess S., Machida K. (2016). NANOG Metabolically Reprograms Tumor-Initiating Stem-like Cells through Tumorigenic Changes in Oxidative Phosphorylation and Fatty Acid Metabolism. Cell Metab..

[117] Praharaj P.P., Patro B.S., Bhutia S.K. (2022). Dysregulation of mitophagy and mitochondrial homeostasis in cancer stem cells: Novel mechanism for anti-cancer stem cell-targeted cancer therapy. Br. J. Pharmacol..

[118] Zhang H., Menzies K.J., Auwerx J. (2018). The role of mitochondria in stem cell fate and aging. Development.

[119] Adams P.D., Jasper H., Rudolph K.L. (2015). Aging-Induced Stem Cell Mutations as Drivers for Disease and Cancer. Cell Stem Cell.

[120] Sell S. (2010). On the stem cell origin of cancer. Am. J. Pathol..

[121] Fan M., Shi Y., Zhao J., Li L. (2023). Cancer stem cell fate determination: Mito-nuclear communication. Cell Commun. Signal..

[122] Guevara-Aguirre J., Balasubramanian P., Guevara-Aguirre M., Wei M., Madia F., Cheng C.W., Hwang D., Martin-Montalvo A., Saavedra J., Ingles S. (2011). Growth hormone receptor deficiency is associated with a major reduction in pro-aging signaling, cancer, and diabetes in humans. Sci. Transl. Med..

[123] Shevah O., Laron Z. (2007). Patients with congenital deficiency of IGF-I seem protected from the development of malignancies: A preliminary report. Growth Horm. IGF Res..

[124] Guevara-Aguirre J., Guevara C., Guevara A., Gavilanes A.A. (2020). Branding of subjects affected with genetic syndromes of severe short stature in developing countries. BMJ Case Rep..

[125] Akbari M., Kirkwood T.B.L., Bohr V.A. (2019). Mitochondria in the signaling pathways that control longevity and health span. Ageing Res. Rev..

[126] Ratajczak J., Shin D.M., Wan W., Liu R., Masternak M.M., Piotrowska K., Wiszniewska B., Kucia M., Bartke A., Ratajczak M.Z. (2011). Higher number of stem cells in the bone marrow of circulating low Igf-1 level Laron dwarf mice--novel view on Igf-1, stem cells and aging. Leukemia.

[127] Brown-Borg H.M., Johnson W.T., Rakoczy S.G. (2012). Expression of oxidative phosphorylation components in mitochondria of long-living Ames dwarf mice. Age.

[128] Chen P.C., Kuo Y.C., Chuong C.M., Huang Y.H. (2020). Niche Modulation of IGF-1R Signaling: Its Role in Stem Cell Pluripotency, Cancer Reprogramming, and Therapeutic Applications. Front. Cell Dev. Biol..

[129] De Francesco E.M., Sotgia F., Lisanti M.P. (2018). Cancer stem cells (CSCs): Metabolic strategies for their identification and eradication. Biochem. J..

[130] Liu Y., Sun Y., Guo Y., Shi X., Chen X., Feng W., Wu L.L., Zhang J., Yu S., Wang Y. (2023). An Overview: The Diversified Role of Mitochondria in Cancer Metabolism. Int. J. Biol. Sci..

[131] Chae H.S., Hong S.T. (2022). Overview of Cancer Metabolism and Signaling Transduction. Int. J. Mol. Sci..

[132] Pavlova N.N., Zhu J., Thompson C.B. (2022). The hallmarks of cancer metabolism: Still emerging. Cell Metab..

[133] Jones C.L., Inguva A., Jordan C.T. (2021). Targeting Energy Metabolism in Cancer Stem Cells: Progress and Challenges in Leukemia and Solid Tumors. Cell Stem Cell.

[134] Garde A., Sherwood D.R. (2021). Fueling Cell Invasion through Extracellular Matrix. Trends. Cell Biol..

[135] Busk M., Horsman M.R., Kristjansen P.E., van der Kogel A.J., Bussink J., Overgaard J. (2008). Aerobic glycolysis in cancers: Implications for the usability of oxygen-responsive genes and fluorodeoxyglucose-PET as markers of tissue hypoxia. Int. J. Cancer.

[136] González M.J., Rosario-Pérez G., Guzmán A.M., Miranda-Massari J.R., Duconge J., Lavergne J., Fernandez N., Ortiz N., Quintero A., Mikirova N. (2010). Mitochondria, Energy and Cancer: The Relationship with Ascorbic Acid. J. Orthomol. Med..

[137] Epstein T., Gatenby R.A., Brown J.S. (2017). The Warburg effect as an adaptation of cancer cells to rapid fluctuations in energy demand. PLoS ONE.

[138] Pfeiffer T., Schuster S., Bonhoeffer S. (2001). Cooperation and competition in the evolution of ATP-producing pathways. Science.

[139] Guevara-Aguirre J., Peña G., Pazmiño G., Acosta W., Saavedra J., Lescano D., Guevara A., Gavilanes A.W.D. (2023). Cancer in Ecuadorian subjects with Laron syndrome (ELS). Endocr. Relat. Cancer.

[140] Lukey M.J., Wilson K.F., Cerione R.A. (2013). Therapeutic strategies impacting cancer cell glutamine metabolism. Future Med. Chem..

[141] Liao J., Liu P.-P., Hou G., Shao J., Yang J., Liu K., Lu W., Wen S., Hu Y., Huang P. (2017). Regulation of stem-like cancer cells by glutamine through β-catenin pathway mediated by redox signaling. Mol. Cancer.

[142] Seyfried T.N., Shelton L.M. (2010). Cancer as a metabolic disease. Nutr. Metab..

[143] Sabnis H.S., Somasagara R.R., Bunting K.D. (2017). Targeting MYC Dependence by Metabolic Inhibitors in Cancer. Genes.

[144] Griffeth L.K. (2005). Use of PET/CT scanning in cancer patients: Technical and practical considerations. Proc. (Bayl. Univ. Med. Cent.).

[145] Zhu L., Ploessl K., Zhou R., Mankoff D., Kung H.F. (2017). Metabolic Imaging of Glutamine in Cancer. J. Nucl. Med..

[146] Zahra K., Dey T., Ashish, Mishra S.P., Pandey U. (2020). Pyruvate Kinase M2 and Cancer: The Role of PKM2 in Promoting Tumorigenesis. Front. Oncol..

[147] Chinopoulos C. (2020). From Glucose to Lactate and Transiting Intermediates Through Mitochondria, Bypassing Pyruvate Kinase: Considerations for Cells Exhibiting Dimeric PKM2 or Otherwise Inhibited Kinase Activity. Front. Physiol..

[148] Persi E., Duran-Frigola M., Damaghi M., Roush W.R., Aloy P., Cleveland J.L., Gillies R.J., Ruppin E. (2018). Systems analysis of intracellular pH vulnerabilities for cancer therapy. Nat. Commun..

[149] Abd G.M., Laird M.C., Ku J.C., Li Y. (2023). Hypoxia-induced cancer cell reprogramming: A review on how cancer stem cells arise. Front. Oncol..

[150] Qian J., Rankin E.B. (2019). Hypoxia-Induced Phenotypes that Mediate Tumor Heterogeneity. Adv. Exp. Med. Biol..

[151] Duraj T., Carrión-Navarro J., Seyfried T.N., García-Romero N., Ayuso-Sacido A. (2021). Metabolic therapy and bioenergetic analysis: The missing piece of the puzzle. Mol. Metab..

[152] Bajzikova M., Kovarova J., Coelho A.R., Boukalova S., Oh S., Rohlenova K., Svec D., Hubackova S., Endaya B., Judasova K. (2019). Reactivation of Dihydroorotate Dehydrogenase-Driven Pyrimidine Biosynthesis Restores Tumor Growth of Respiration-Deficient Cancer Cells. Cell Metab..

[153] Ta N.L., Seyfried T.N. (2015). Influence of Serum and Hypoxia on Incorporation of [(14)C]-D-Glucose or [(14)C]-L-Glutamine into Lipids and Lactate in Murine Glioblastoma Cells. Lipids.

[154] Renner C., Asperger A., Seyffarth A., Meixensberger J., Gebhardt R., Gaunitz F. (2010). Carnosine inhibits ATP production in cells from malignant glioma. Neurol. Res..

[155] Barron E.S. (1930). The catalytic effect of methylene blue on the oxygen consumption of tumors and normal tissues. J. Exp. Med..

[156] Warburg O., Wind F., Negelein E. (1927). The metabolism of tumors in the body. J. Gen. Physiol..

[157] Ceruti S., Mazzola A., Abbracchio M.P. (2005). Resistance of human astrocytoma cells to apoptosis induced by mitochondria-damaging agents: Possible implications for anticancer therapy. J. Pharmacol. Exp. Ther..

[158] Pastò A., Bellio C., Pilotto G., Ciminale V., Silic-Benussi M., Guzzo G., Rasola A., Frasson C., Nardo G., Zulato E. (2014). Cancer stem cells from epithelial ovarian cancer patients privilege oxidative phosphorylation, and resist glucose deprivation. Oncotarget.

[159] DeBerardinis R.J., Chandel N.S. (2016). Fundamentals of cancer metabolism. Sci. Adv..

[160] Mathews E.H., Stander B.A., Joubert A.M., Liebenberg L. (2014). Tumor cell culture survival following glucose and glutamine deprivation at typical physiological concentrations. Nutrition.

[161] Holm E., Hagmüller E., Staedt U., Schlickeiser G., Günther H., Leweling H., Tokus M., Kollmar H. (1995). Substrate balances across colonic carcinomas in Humans. Cancer Res..

[162] Lawson D.A., Bhakta N.R., Kessenbrock K., Prummel K.D., Yu Y., Takai K., Zhou A., Eyob H., Balakrishnan S., Wang C.Y. (2015). Single-cell analysis reveals a stem-cell program in human metastatic breast cancer cells. Nature.

[163] Song Q., Ruiz J., Xing F., Lo H.W., Craddock L., Pullikuth A.K., Miller L.D., Soike M.H., O’Neill S.S., Watabe K. (2023). Single-cell sequencing reveals the landscape of the human brain metastatic microenvironment. Commun. Biol..

[164] Li R., Liu X., Huang X., Zhang D., Chen Z., Zhang J., Bai R., Zhang S., Zhao H., Xu Z. (2024). Single-cell transcriptomic analysis deciphers heterogenous cancer stem-like cells in colorectal cancer and their organ-specific metastasis. Gut.

[165] Pan X.W., Zhang H., Xu D., Chen J.X., Chen W.J., Gan S.S., Qu F.J., Chu C.M., Cao J.W., Fan Y.H. (2020). Identification of a novel cancer stem cell subpopulation that promotes progression of human fatal renal cell carcinoma by single-cell RNA-seq analysis. Int. J. Biol. Sci..

[166] Baccelli I., Trumpp A. (2012). The evolving concept of cancer and metastasis stem cells. J. Cell Biol..

[167] Verga Falzacappa M.V., Ronchini C., Reavie L.B., Pelicci P.G. (2012). Regulation of self-renewal in normal and cancer stem cells. FEBS J..

[168] Lambert A.W., Pattabiraman D.R., Weinberg R.A. (2017). Emerging Biological Principles of Metastasis. Cell.

[169] Steinbichler T.B., Savic D., Dudás J., Kvitsaridze I., Skvortsov S., Riechelmann H., Skvortsova I.I. (2020). Cancer stem cells and their unique role in metastatic spread. Semin Cancer Biol..

[170] Shiozawa Y., Nie B., Pienta K.J., Morgan T.M., Taichman R.S. (2013). Cancer stem cells and their role in metastasis. Pharmacol. Ther..

[171] Seyfried T.N., Huysentruyt L.C. (2013). On the origin of cancer metastasis. Crit. Rev. Oncog..

[172] Aguirre L.A., Montalbán-Hernández K., Avendaño-Ortiz J., Marín E., Lozano R., Toledano V., Sánchez-Maroto L., Terrón V., Valentín J., Pulido E. (2020). Tumor stem cells fuse with monocytes to form highly invasive tumor-hybrid cells. Oncoimmunology.

[173] Solinas G., Germano G., Mantovani A., Allavena P. (2009). Tumor-associated macrophages (TAM) as major players of the cancer-related inflammation. J. Leukoc. Biol..

[174] Aramini B., Masciale V., Grisendi G., Banchelli F., D’Amico R., Maiorana A., Morandi U., Dominici M., Haider K.H. (2021). Cancer stem cells and macrophages: Molecular connections and future perspectives against cancer. Oncotarget.

[175] Allavena P., Digifico E., Belgiovine C. (2021). Macrophages and cancer stem cells: A malevolent alliance. Mol. Med..

[176] Sharma V.P., Tang B., Wang Y., Duran C.L., Karagiannis G.S., Xue E.A., Entenberg D., Borriello L., Coste A., Eddy R.J. (2021). Live tumor imaging shows macrophage induction and TMEM-mediated enrichment of cancer stem cells during metastatic dissemination. Nat. Commun..

[177] Chaffer C.L., Brueckmann I., Scheel C., Kaestli A.J., Wiggins P.A., Rodrigues L.O., Brooks M., Reinhardt F., Su Y., Polyak K. (2011). Normal and neoplastic nonstem cells can spontaneously convert to a stem-like state. Proc. Natl. Acad. Sci. USA.

[178] Tang D.G. (2012). Understanding cancer stem cell heterogeneity and plasticity. Cell Res..

[179] Alibert C., Goud B., Manneville J.B. (2017). Are cancer cells really softer than normal cells?. Biol. Cell.

[180] Burgdorf S., Porubsky S., Marx A., Popovic Z.V. (2020). Cancer Acidity and Hypertonicity Contribute to Dysfunction of Tumor-Associated Dendritic Cells: Potential Impact on Antigen Cross-Presentation Machinery. Cancers.

[181] Follain G., Herrmann D., Harlepp S., Hyenne V., Osmani N., Warren S.C., Timpson P., Goetz J.G. (2020). Fluids and their mechanics in tumour transit: Shaping metastasis. Nat. Rev. Cancer.

[182] Wu M., Frieboes H.B., McDougall S.R., Chaplain M.A., Cristini V., Lowengrub J. (2013). The effect of interstitial pressure on tumor growth: Coupling with the blood and lymphatic vascular systems. J. Theor. Biol..

[183] Devic S. (2016). Warburg Effect—A Consequence or the Cause of Carcinogenesis?. J. Cancer.

[184] Morita M., Sato T., Nomura M., Sakamoto Y., Inoue Y., Tanaka R., Ito S., Kurosawa K., Yamaguchi K., Sugiura Y. (2018). PKM1 Confers Metabolic Advantages and Promotes Cell-Autonomous Tumor Cell Growth. Cancer Cell.

[185] Marchiq I., Pouysségur J. (2016). Hypoxia, cancer metabolism and the therapeutic benefit of targeting lactate/H(+) symporters. J. Mol. Med..

[186] Seyfried T.N., Arismendi-Morillo G., Zuccoli G., Lee D.C., Duraj T., Elsakka A.M., Maroon J.C., Mukherjee P., Ta L., Shelton L. (2022). Metabolic management of microenvironment acidity in glioblastoma. Front. Oncol..

[187] Farhana A., Alsrhani A., Khan Y.S., Rasheed Z. (2023). Cancer Bioenergetics and Tumor Microenvironments—Enhancing Chemotherapeutics and Targeting Resistant Niches through Nanosystems. Cancers.

[188] da Veiga Moreira J., De Staercke L., César Martínez-Basilio P., Gauthier-Thibodeau S., Montégut L., Schwartz L., Jolicoeur M. (2021). Hyperosmolarity Triggers the Warburg Effect in Chinese Hamster Ovary Cells and Reveals a Reduced Mitochondria Horsepower. Metabolites.

[189] San-Millán I., Brooks G.A. (2017). Reexamining cancer metabolism: Lactate production for carcinogenesis could be the purpose and explanation of the Warburg Effect. Carcinogenesis.

[190] Choi S.Y., Collins C.C., Gout P.W., Wang Y. (2013). Cancer-generated lactic acid: A regulatory, immunosuppressive metabolite?. J. Pathol..

[191] Schwartz L., Supuran C.T., Alfarouk K.O. (2017). The Warburg Effect and the Hallmarks of Cancer. Anticancer Agents Med. Chem..

[192] Cardone R.A., Casavola V., Reshkin S.J. (2005). The role of disturbed pH dynamics and the Na^+^/H^+^ exchanger in metastasis. Nat. Rev. Cancer.

[193] Gallagher F.A., Kettunen M.I., Day S.E., Hu D.-E., Ardenkjær-Larsen J.H., Zandt R.i.t., Jensen P.R., Karlsson M., Golman K., Lerche M.H. (2008). Magnetic resonance imaging of pH in vivo using hyperpolarized 13C-labelled bicarbonate. Nature.

[194] Gillies R.J., Raghunand N., Karczmar G.S., Bhujwalla Z.M. (2002). MRI of the tumor microenvironment. J. Magn. Reson. Imaging.

[195] Chiche J., Brahimi-Horn M.C., Pouysségur J. (2010). Tumour hypoxia induces a metabolic shift causing acidosis: A common feature in cancer. J. Cell. Mol. Med..

[196] Wang Y.H., Scadden D.T. (2015). Targeting the Warburg effect for leukemia therapy: Magnitude matters. Mol. Cell. Oncol..

[197] Gregory M.A., Nemkov T., Park H.J., Zaberezhnyy V., Gehrke S., Adane B., Jordan C.T., Hansen K.C., D’Alessandro A., DeGregori J. (2019). Targeting Glutamine Metabolism and Redox State for Leukemia Therapy. Clin. Cancer Res..

[198] Shanmugam M., McBrayer S.K., Rosen S.T. (2009). Targeting the Warburg effect in hematological malignancies: From PET to therapy. Curr. Opin. Oncol..

[199] Ma S., Lee H., Jo W.Y., Byun Y.H., Shin K.W., Choi S., Oh H., Park C.K., Park H.P. (2023). The Warburg effect in patients with brain tumors: A comprehensive analysis of clinical significance. J. Neurooncol..

[200] Chen Z., Han F., Du Y., Shi H., Zhou W. (2023). Hypoxic microenvironment in cancer: Molecular mechanisms and therapeutic interventions. Signal Transduct. Target. Ther..

[201] Vito A., El-Sayes N., Mossman K. (2020). Hypoxia-Driven Immune Escape in the Tumor Microenvironment. Cells.

[202] Kabakov A.E., Yakimova A.O. (2021). Hypoxia-Induced Cancer Cell Responses Driving Radioresistance of Hypoxic Tumors: Approaches to Targeting and Radiosensitizing. Cancers.

[203] Finley L.W.S., Thompson C.B., Mendelsohn J., Gray J.W., Howley P.M., Israel M.A., Thompson C.B. (2015). 13—The Metabolism of Cell Growth and Proliferation. The Molecular Basis of Cancer.

[204] Vaupel P., Multhoff G. (2018). Hypoxia-/HIF-1α-Driven Factors of the Tumor Microenvironment Impeding Antitumor Immune Responses and Promoting Malignant Progression. Adv. Exp. Med. Biol..

[205] Nijman S.M.B. (2020). Perturbation-Driven Entropy as a Source of Cancer Cell Heterogeneity. Trends Cancer.

[206] Hanselmann R.G., Welter C. (2016). Origin of Cancer: An Information, Energy, and Matter Disease. Front. Cell. Dev. Biol..

[207] Knapp J.P., Kakish J.E., Bridle B.W., Speicher D.J. (2022). Tumor Temperature: Friend or Foe of Virus-Based Cancer Immunotherapy. Biomedicines.

[208] El-Gammal Z., Nasr M.A., Elmehrath A.O., Salah R.A., Saad S.M., El-Badri N. (2022). Regulation of mitochondrial temperature in health and disease. Pflügers Arch.—Eur. J. Physiol..

[209] Werb Z., Lu P. (2015). The Role of Stroma in Tumor Development. Cancer J..

[210] Baker S.G., Kramer B.S. (2007). Paradoxes in carcinogenesis: New opportunities for research directions. BMC Cancer.

[211] Robinson A.J., Jain A., Sherman H.G., Hague R.J.M., Rahman R., Sanjuan-Alberte P., Rawson F.J. (2021). Toward Hijacking Bioelectricity in Cancer to Develop New Bioelectronic Medicine. Adv. Ther..

[212] Tiwari B.S., Belenghi B., Levine A. (2002). Oxidative stress increased respiration and generation of reactive oxygen species, resulting in ATP depletion, opening of mitochondrial permeability transition, and programmed cell death. Plant Physiol..

[213] Pokorný J., Pokorný J., Borodavka F. (2017). Warburg effect—Damping of electromagnetic oscillations. Electromagn. Biol. Med..

[214] Pokorný J., Pokorný J., Kobilková J., Jandová A., Holaj R. (2020). Cancer Development and Damped Electromagnetic Activity. Appl. Sci..

[215] Behnam B., Taghizadeh-Hesary F. (2023). Mitochondrial Metabolism: A New Dimension of Personalized Oncology. Cancers.

[216] Bagchi B. (2013). Dynamics of water: Molecular motions and hydrogen-bond-breaking kinetics. Water in Biological and Chemical Processes: From Structure and Dynamics to Function.

[217] Marques M.P.M., Batista de Carvalho A.L.M., Mamede A.P., Dopplapudi A., García Sakai V., Batista de Carvalho L.A.E. (2020). Role of intracellular water in the normal-to-cancer transition in human cells-insights from quasi-elastic neutron scattering. Struct. Dyn..

[218] Tanner K., Mori H., Mroue R., Bruni-Cardoso A., Bissell M.J. (2012). Coherent angular motion in the establishment of multicellular architecture of glandular tissues. Proc. Natl. Acad. Sci. USA.

[219] de Visser K.E., Joyce J.A. (2023). The evolving tumor microenvironment: From cancer initiation to metastatic outgrowth. Cancer Cell.

[220] Lee C.H., Cho J., Lee K. (2020). Tumour Regression via Integrative Regulation of Neurological, Inflammatory, and Hypoxic Tumour Microenvironment. Biomol. Ther..

[221] Scheid A.D., Beadnell T.C., Welch D.R. (2021). Roles of mitochondria in the hallmarks of metastasis. Br. J. Cancer.

[222] Song I.S., Jeong J.Y., Jeong S.H., Kim H.K., Ko K.S., Rhee B.D., Kim N., Han J. (2015). Mitochondria as therapeutic targets for cancer stem cells. World J. Stem Cells.

[223] Taghizadeh-Hesary F., Akbari H., Bahadori M., Behnam B. (2022). Targeted Anti-Mitochondrial Therapy: The Future of Oncology. Genes.

[224] Missiroli S., Perrone M., Genovese I., Pinton P., Giorgi C. (2020). Cancer metabolism and mitochondria: Finding novel mechanisms to fight tumours. eBioMedicine.

[225] Duan Z., Luo Y. (2021). Targeting macrophages in cancer immunotherapy. Signal Transduct. Target. Ther..

[226] Sia J., Szmyd R., Hau E., Gee H.E. (2020). Molecular Mechanisms of Radiation-Induced Cancer Cell Death: A Primer. Front. Cell. Dev. Biol..

[227] van den Boogaard W.M.C., Komninos D.S.J., Vermeij W.P. (2022). Chemotherapy Side-Effects: Not All DNA Damage Is Equal. Cancers.

[228] Min H.Y., Lee H.Y. (2022). Molecular targeted therapy for anticancer treatment. Exp. Mol. Med..

[229] Iwasaki H., Suda T. (2009). Cancer stem cells and their niche. Cancer Sci..

[230] Gerstung M., Jolly C., Leshchiner I., Dentro S.C., Gonzalez S., Rosebrock D., Mitchell T.J., Rubanova Y., Anur P., Yu K. (2020). The evolutionary history of 2658 cancers. Nature.

[231] Lytle N.K., Barber A.G., Reya T. (2018). Stem cell fate in cancer growth, progression and therapy resistance. Nat. Rev. Cancer.

[232] Phi L.T.H., Sari I.N., Yang Y.G., Lee S.H., Jun N., Kim K.S., Lee Y.K., Kwon H.Y. (2018). Cancer Stem Cells (CSCs) in Drug Resistance and their Therapeutic Implications in Cancer Treatment. Stem Cells Int..

[233] Ansooya B., Patricia S. (2019). Mitochondrial determinants of chemoresistance. Mitochondrial Determ. Chemoresistance.

[234] García-Heredia J.M., Carnero A. (2020). Role of Mitochondria in Cancer Stem Cell Resistance. Cells.

[235] Pellicano F., Mukherjee L., Holyoake T.L. (2014). Concise review: Cancer cells escape from oncogene addiction: Understanding the mechanisms behind treatment failure for more effective targeting. Stem Cells.

[236] Jiang X., Saw K.M., Eaves A., Eaves C. (2007). Instability of BCR-ABL gene in primary and cultured chronic myeloid leukemia stem cells. J. Natl. Cancer Inst..

[237] Ladanie A., Schmitt A.M., Speich B., Naudet F., Agarwal A., Pereira T.V., Sclafani F., Herbrand A.K., Briel M., Martin-Liberal J. (2020). Clinical Trial Evidence Supporting US Food and Drug Administration Approval of Novel Cancer Therapies Between 2000 and 2016. JAMA Netw. Open.

[238] Del Paggio J.C., Berry J.S., Hopman W.M., Eisenhauer E.A., Prasad V., Gyawali B., Booth C.M. (2021). Evolution of the Randomized Clinical Trial in the Era of Precision Oncology. JAMA Oncol..

[239] Gorini S., De Angelis A., Berrino L., Malara N., Rosano G., Ferraro E. (2018). Chemotherapeutic Drugs and Mitochondrial Dysfunction: Focus on Doxorubicin, Trastuzumab, and Sunitinib. Oxid. Med. Cell. Longev..

[240] Averbeck D., Rodriguez-Lafrasse C. (2021). Role of Mitochondria in Radiation Responses: Epigenetic, Metabolic, and Signaling Impacts. Int. J. Mol. Sci..

[241] Cohen J.B., Brown N.J., Brown S.A., Dent S., van Dorst D.C.H., Herrmann S.M., Lang N.N., Oudit G.Y., Touyz R.M. (2023). Cancer Therapy-Related Hypertension: A Scientific Statement From the American Heart Association. Hypertension.

[242] Bikomeye J.C., Terwoord J.D., Santos J.H., Beyer A.M. (2022). Emerging mitochondrial signaling mechanisms in cardio-oncology: Beyond oxidative stress. Am. J. Physiol. Heart Circ. Physiol..

[243] Correia A.L., Bissell M.J. (2012). The tumor microenvironment is a dominant force in multidrug resistance. Drug Resist. Updat..

[244] Xiong W., Liao Y., Qin J.Y., Li W.H., Tang Z.Y. (2020). Adverse effects of chemoradiotherapy on invasion and metastasis of tumor cells. Genes Dis..

[245] Ratajczak M.Z., Bujko K., Mack A., Kucia M., Ratajczak J. (2018). Cancer from the perspective of stem cells and misappropriated tissue regeneration mechanisms. Leukemia.

[246] Miller K.D., Nogueira L., Mariotto A.B., Rowland J.H., Yabroff K.R., Alfano C.M., Jemal A., Kramer J.L., Siegel R.L. (2019). Cancer treatment and survivorship statistics, 2019. CA Cancer J. Clin..

[247] Miller K.D., Nogueira L., Devasia T., Mariotto A.B., Yabroff K.R., Jemal A., Kramer J., Siegel R.L. (2022). Cancer treatment and survivorship statistics, 2022. CA Cancer J. Clin..

[248] Vincent A., Herman J., Schulick R., Hruban R.H., Goggins M. (2011). Pancreatic cancer. Lancet.

[249] Chaffer C.L., Weinberg R.A. (2011). A perspective on cancer cell metastasis. Science.

[250] Jin P., Jiang J., Zhou L., Huang Z., Nice E.C., Huang C., Fu L. (2022). Mitochondrial adaptation in cancer drug resistance: Prevalence, mechanisms, and management. J. Hematol. Oncol..

[251] Bao B., Ahmad A., Azmi A.S., Ali S., Sarkar F.H. (2013). Overview of cancer stem cells (CSCs) and mechanisms of their regulation: Implications for cancer therapy. Curr. Protoc. Pharmacol..

[252] Morrison B.J., Steel J.C., Morris J.C. (2018). Reduction of MHC-I expression limits T-lymphocyte-mediated killing of Cancer-initiating cells. BMC Cancer.

[253] Larionova I., Cherdyntseva N., Liu T., Patysheva M., Rakina M., Kzhyshkowska J. (2019). Interaction of tumor-associated macrophages and cancer chemotherapy. Oncoimmunology.

[254] Galluzzi L., Buqué A., Kepp O., Zitvogel L., Kroemer G. (2015). Immunological Effects of Conventional Chemotherapy and Targeted Anticancer Agents. Cancer Cell.

[255] Moradi Kashkooli F., Soltani M. (2021). Evaluation of solid tumor response to sequential treatment cycles via a new computational hybrid approach. Sci. Rep..

[256] Erdi Y.E. (2012). Limits of Tumor Detectability in Nuclear Medicine and PET. Mol. Imaging Radionucl. Ther..

[257] Narod S.A. (2012). Disappearing breast cancers. Curr. Oncol..

[258] Fischer B.M., Olsen M.W., Ley C.D., Klausen T.L., Mortensen J., Højgaard L., Kristjansen P.E. (2006). How few cancer cells can be detected by positron emission tomography? A frequent question addressed by an in vitro study. Eur. J. Nucl. Med. Mol. Imaging.

[259] Zhou H.-M., Zhang J.-G., Zhang X., Li Q. (2021). Targeting cancer stem cells for reversing therapy resistance: Mechanism, signaling, and prospective agents. Signal Transduct. Target. Ther..

[260] Zheng X.X., Chen J.J., Sun Y.B., Chen T.Q., Wang J., Yu S.C. (2023). Mitochondria in cancer stem cells: Achilles heel or hard armor. Trends Cell Biol..

[261] Vuda M., Kamath A. (2016). Drug induced mitochondrial dysfunction: Mechanisms and adverse clinical consequences. Mitochondrion.

[262] Fromenty B., Pessayre D. (1995). Inhibition of mitochondrial beta-oxidation as a mechanism of hepatotoxicity. Pharmacol. Ther..

[263] Moen I., Stuhr L.E. (2012). Hyperbaric oxygen therapy and cancer—A review. Target. Oncol..

[264] Bianchi G., Martella R., Ravera S., Marini C., Capitanio S., Orengo A., Emionite L., Lavarello C., Amaro A., Petretto A. (2015). Fasting induces anti-Warburg effect that increases respiration but reduces ATP-synthesis to promote apoptosis in colon cancer models. Oncotarget.

